# Experimental constraints on pre-eruptive conditions at Nabro volcano, Eritrea, 2011, and implications for sulphur emissions

**DOI:** 10.1007/s00410-026-02357-2

**Published:** 2026-09-04

**Authors:** Amy Donovan, Joan Andújar, Bruno Scaillet, Iris Buisman, Clive Oppenheimer

**Affiliations:** 1 Department of Geography, University of Cambridge, Downing Place, Cambridge, CB2 3EN UK; 2 Institut des Sciences de la Terre d’Orleans, UMR 7327, Université d’Orleans-CNRS‐BRGM, Orleans, France; 3 Department of Earth Sciences, University of Cambridge, Downing Street, Cambridge, CB2 3EQ UK; 4 Istituto Nazionale di Geofisica e Vulcanologia, Osservatorio Etneo, Piazza Roma, 2, 95125 Catania, Italy

**Keywords:** Nabro, Volcanic gas emission, Experimental petrology, Phase equilibria, Excess fluid

## Abstract

We present new experimental constraints on the pre-eruptive magma storage conditions at Nabro Volcano, Eritrea, prior to its 2011 eruption, and discuss the implications for sulphur emissions. We find that the magma was likely stored in the shallow crust prior to eruption, following an intrusion in March 2011. This reservoir was located at ~ 7 km depth, and was recharged immediately prior to the eruption, from a deeper reservoir located at 20 km. The temperature of the magma was 1030 °C with 3 wt% dissolved H_2_O, at redox conditions around the Quartz-Fayalite-Magnetite buffer (QFM). Our experimental results show that most melt inclusions from previous studies record earlier stages of magmatic evolution. Based on the determined pre-eruptive conditions, we calculate the composition and amount of pre-eruptive excess fluid and find that 3–8 wt% of an excess fluid with 1–3 wt% S is needed to explain the amount of SO_2_ measured by satellite remote sensing. The abundance of this excess fluid may have contributed to the explosive character of the eruption, whose plume reached the lower stratosphere, assisted by convective transport linked to the Asian monsoon.

**Supplementary Information:**

The online version contains supplementary material available at https://doi.org/10.1007/s00410-026-02357-2.

## Introduction

The production of large volumes of SO_2_ in large basaltic eruptions is relatively rare, yet may have considerable impact on the climate system (Longpré et al. 2017; Oppenheimer et al. 2011; Schmidt et al. 2016). Furthermore, the complexity of sulphur speciation in magmas creates high uncertainties around the production and potential storage of gas at different depths in the plumbing system (Scaillet and Oppenheimer 2023, 2024). Recent eruptions in La Palma and Iceland have, however, demonstrated the importance of understanding sulphur systematics and the presence or absence of excess sulphur in magmatic reservoirs prior to eruption (Esse et al. 2023; Fabbrizio et al. 2023; Frascerra et al. 2024; Pfeffer et al. 2024; Taquet et al. 2025).

Magmatic bodies in the crust may contain considerable volumes of compressed gas (e.g., Kilbride et al. 2016). Calculating the fraction of gas requires knowledge of intrinsic variables such as pressure, temperature, oxygen fugacity (fO_2_) and composition. Petrological studies of melt inclusions are one method that may be used to provide information, based on assumptions around the partitioning behaviour of volatile species (e.g. Métrich and Wallace 2008; Moore et al. 2015; Scaillet and Oppenheimer 2024). Experimental studies of erupted products may also be used to refine models of pre-eruptive magma bodies and to assess the solubility of volatiles in melts under a range of crustal conditions (e.g. Iacovino et al. 2013; Lesne et al. 2011b, 2015). Satellite and ground-based remote sensing methods allow the measurement of volatiles in volcanic plumes, though are limited by field and instrumental constraints and may require multiple techniques to measure a range of gas species (Oppenheimer et al. 2011; Theys et al. 2013). Each of these methods provides information about the magmatic system and its interaction with the atmosphere; however, each is also based on a set of assumptions that affect data interpretation (Scaillet and Oppenheimer 2024). In this paper, we combine information from melt inclusions and phase equilibrium experiments to investigate the 2011 explosive basaltic eruption at Nabro volcano, Eritrea (Goitom et al. 2015). In doing so, we refine existing models of the pre-eruptive processes that led to the eruption, and provide constraints on the pre-eruptive volatile content of the magma.

## Geological background

**Fig. 1:**
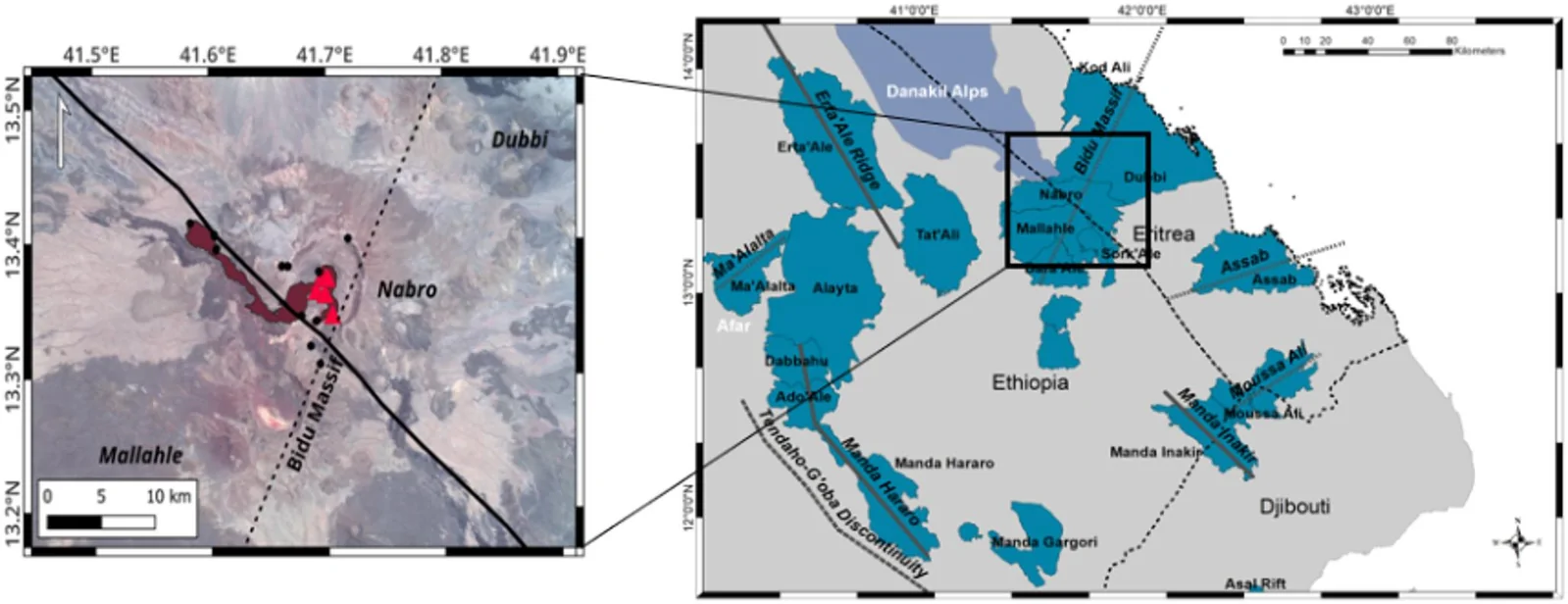
(Left) Eruption setting (lava from 2011 in maroon and vents in red) and location of the samples (black dots). The black dotted line shows trend of the Bidu volcanic range. The thick black line is the border between Ethiopia to SW and Eritrea to NE. Data from Landsat 8. (Right) Tectonic setting of Nabro and the Bidu Massif in the Afar region. Volcanic fields are shown in turquoise, and the Danakil Alps in blue. Spreading rifts are marked with solid grey lines, international borders are black dashed lines and other tectonic lineaments are grey dashed lines

Nabro is a complex stratovolcano in Eritrea, whose flanks extend across the border with Ethiopia (Fig. 1). Its summit hosts an 8-km-diameter caldera, and the surrounding plains are covered by extensive late Quaternary ignimbrites (Oppenheimer et al. 2019). The volcano forms part of the Bidu Massif, described as a “marginal unit” (Barberi et al. 1974a, b) on the edge of the Afar depression. This linear volcanic chain runs from Sork’Ale in the southwest to Kod Ali island in the northeast (Fig. 1). Petrological studies (Donovan et al. 2018; Wiart and Oppenheimer 2005) suggest affinities between Nabro and neighbouring Dubbi volcano, whose products are considerably more alkaline than those of the Erta ‘Ale range that lies roughly 100 km to the northwest of Nabro.

In 2011, Nabro erupted for the first time in recorded history (Goitom et al. 2015). The eruption appears to have been triggered by a fresh basaltic intrusion that remobilised a recently emplaced sill (Donovan et al. 2018). It was fed by a dike that propagated initially NW-SE, but later may have shifted the stress pattern towards NE-SW (Goitom et al. 2015; Hamiel and Baer 2016; Hamlyn et al. 2014). It produced several trachybasaltic lava flows and a substantial tephra field across the Ethiopia–Eritrea border. Thousands of people were displaced (Goitom et al. 2015; Yirgu et al. 2014; Oppenheimer et al. 2019), and the eruption was accompanied by emission of an estimated 4.5 Tg SO_2_ (Bourassa et al. 2012), representing one of the largest sulphur outputs to the atmosphere since Pinatubo’s eruption in 1991.

Aside from some well-studied systems, such as Mount Etna (Alletti et al. 2009; Métrich and Rutherford 1998; Mollo et al. 2017; Vetere et al. 2014), experimental studies on volatile solubility and phase equilibria in alkali basalts are relatively scarce (Freise et al. 2009; Iacovino et al. 2016; Lesne et al. 2011a, b; Sauvalle et al. 2024). Furthermore phase equilibrium models such as MELTS (Ghiorso and Sack 1995) struggle to reproduce phase equilibria of alkali-rich mafic magmas because of a paucity of calibration data in the composition space of these magmas (e.g. Bonechi et al. 2019, 2017; Cristina et al. 2019; Donovan et al. 2018; Fabbrizio et al. 2023; Freise et al. 2009; Rooney et al. 2012). A high alkali content of basaltic melts also strongly impacts CO_2_ solubility (Dixon 1997; Behrens et al. 2009; Jiménez-Mejías et al. 2021; Lesne et al. 2011b; Shishkina et al. 2014; Iacovino et al. 2013), with implications for geobarometric inferences based on melt inclusion volatile contents. Alkali basalts from the margins of the Afar rift have been studied geologically but not experimentally (Barberi et al. 1974a, b; Bizouard et al. 1980; Field et al. 2012, 2013; Moore et al. 2019; Wiart and Oppenheimer 2005). This study aims at filling this gap via experiments on Nabro’s 2011 magma to determine pre-eruption storage conditions. Particular attention was given to determining the $\:{\mathrm{H}}_{2}\mathrm{O}$ and $\:{\mathrm{CO}}_{2}$ contents of residual glasses from experimental charges, using the same analytical conditions and instrument as those used for melt inclusions from that eruption (Donovan et al. 2018). This provides refined constraints on the storage conditions of the 2011 Nabro eruption and highlights the limitations of relying solely on melt inclusions to reconstruct magma volatile budgets. Consequently, we reassess the volatile budget of the Nabro magma and discuss its implications for the high atmospheric sulphur yield.

## Previous work on Nabro

**Fig. 2:**
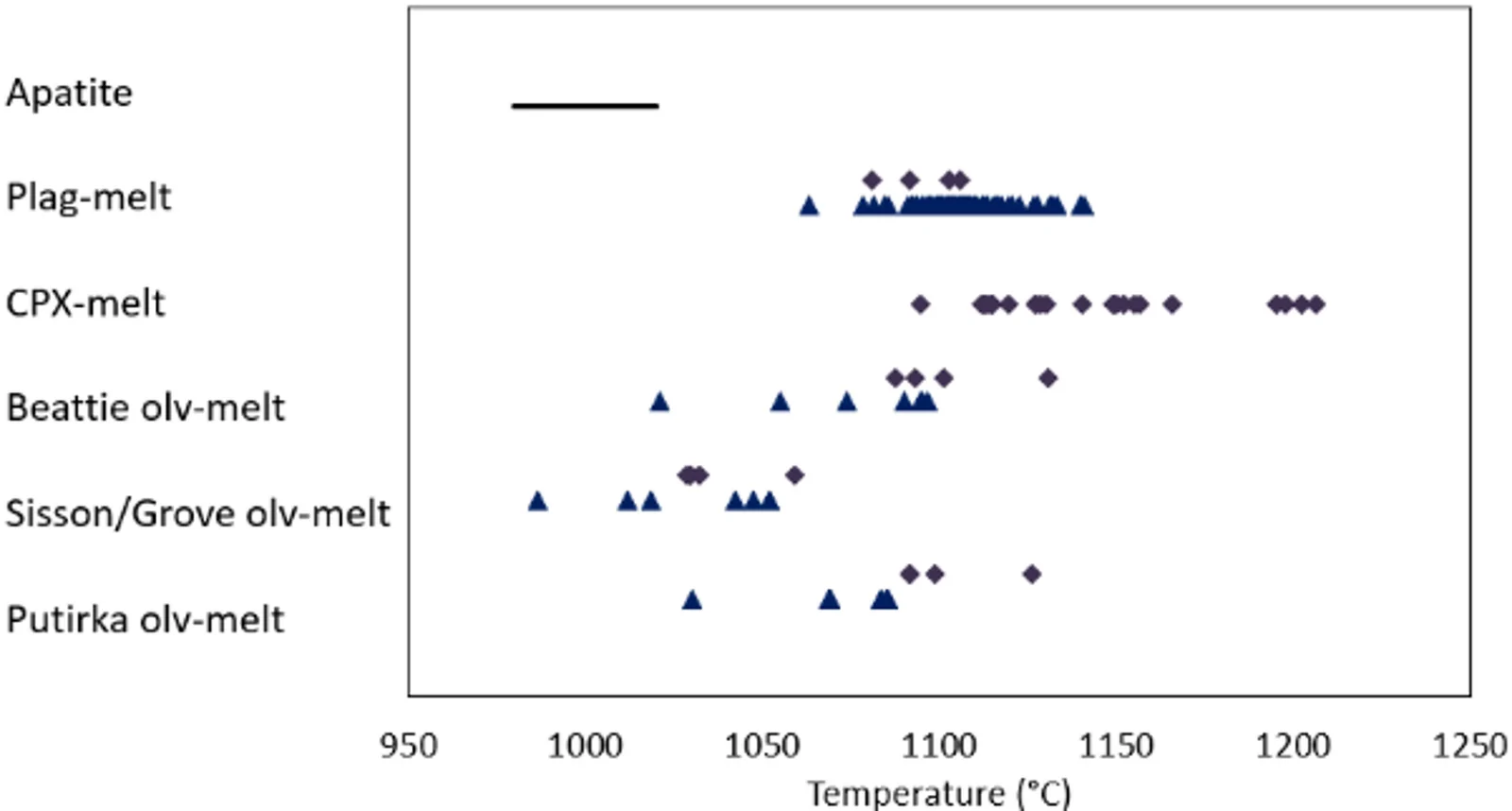
Data from Donovan et al. (2018) for different geothermometers (Beattie 1993; Harrison and Watson 1984; Putirka 2008; Putirka et al. 2003; Sisson and Grove 1993). Diamonds show matrix glass-crystal rims, and triangles are melt inclusions. Calculations performed in Thermobar (Wieser et al. 2022)

This study builds on previous work undertaken at Nabro. The 2011 eruption produced a trachybasaltic magma that was likely intruded in two episodes – one in March 2011 and the other shortly before the eruption (Donovan et al. 2018). These are represented by two distinct textures in the 2011 products, corresponding to batch 1 (glassy, mostly plagioclase both in microlites and phenocrysts with some clinopyroxene as phenocrysts and microlites, and Fe-Ti oxide microlites) and batch 2 (> 70% crystals, again dominated by plagioclase, with clinopyroxene, olivine and Fe-Ti oxides as phenocrysts and as microlites). The bulk chemistry of the two batches is very similar, but batch 1 is slightly richer in Al_2_O_3_ and MgO, and poorer in P_2_O_5_, than the more crystalline batch 2. Melt inclusion studies (Donovan et al. 2018) showed that the magma likely originated in a mush zone at 5–10 km depth beneath the volcano, in which the glassy magma (batch 1) encountered a partly crystalline sill and remobilised it. The range of depths represented in the sample indicates a vertically extensive magmatic system under the volcano, such as inferred for other systems (Cashman et al. 2017). This is also indicated in thermobarometry (Fig. 2). The application of different geothermobarometers (Cpx-liq, plg-liq, apatite, olv-melt) and Ilm-magnetite pairs yield a relatively large range of temperature conditions for the magmas (i.e., 980–1120 °C). Melt inclusion analysis retrieved H_2_O contents of 0.5 to 2.1 wt% (average of 1.5 wt%) and CO_2_ contents of up to 3500 ppm. These volatile contents were converted into entrapment pressures of 20 to up to 500 MPa, most of them clustering at 150 ± 50 MPa and 300 MPa for batch 2 and 1 respectively. The compositions of melt inclusions were distinct from those of the whole rock, having lower SiO_2_ and Al_2_O_3_, and higher FeO*, MgO and TiO_2_. This may reflect late crystallization and accumulation of plagioclase.

Details of phenocryst and microlite compositions are provided in supplementary information. In summary, by combining the textural, petrological and chemical information obtained from the natural products, we consider that the last equilibration episode experience by the magma prior to the eruption is represented by the rim crystallization of An50-60 Plagioclase, Fo70 Olivine, En 40–43 Clinopyroxene, Mt-Ilm and Ap.

This study seeks to constrain further the intrinsic variables represented in the magmas – particularly the location of the sill that fed the 2011 eruption (i.e. batch 2), likely represented by the rim compositions above. It does this using phase equilibrium experiments on a volcanic bomb from batch 2, and then combines data from this study and Donovan et al. (2018) to propose a degassing model for the 2011 eruption.

## Methods

EPMA analysis was done at the University of Cambridge. XRF analysis was done at CNRS-Nancy, and experimental work, including sample preparation, was done at the Institut du Sciences de la Terre d’Orléans. Ion microprobe analysis was undertaken at the Edinburgh Ion Microprobe Facility (EIMF).

### Starting material

The selected starting material is a trachybasaltic lava bomb sample from the 2011 eruption, which is phenocryst-poor, with olivine (Ol), clinopyroxene (Cpx), plagioclase (Pl), magnetite (Mt), ilmenite (Ilm), apatite (Ap) microlites, in addition to residual glass (Gl), and representative of Donovan et al. (2018)’s “batch 2” (Table 1). Additional details on phenocryst zoning and rim compositions are given in the SI. The lava bomb was chosen because it is pristine, geochemically representative of the 2011 tephra samples and contains few phenocrysts, suggesting that it is an example of the dominant melt composition for the magma feeding this eruption.

Several pieces of the lava bomb were crushed in an agate mortar to a fine powder that was melted at 1400 °C in an open atmosphere furnace for 5 h, following a two-step process with grinding in between. The composition of the resulting glass, as determined by electron microprobe microanalyses (EPMA), is similar to the XRF analysis of the bulk rock (Table 1). The dry glass was then ground, and the resulting powder was used as starting material for the phase equilibrium runs. After use, the powder was kept desiccated in an oven at 120 °C.

**Table 1:** Composition of the bulk rock and the starting glass

	Bulk rock (XRF)	Starting material
*N*		6
SiO_2_	48.11	47.51
TiO_2_	3.20	3.26
Al_2_O_3_	15.65	15.24
FeO_T_	11.07	11.00
MnO	0.204	0.22
MgO	5.10	4.96
CaO	8.94	8.67
Na_2_O	4.07	4.06
K_2_O	1.355	1.43
P_2_O_5_	0.994	1.03
S (ppm)	-	758
Cl (ppm)	-	1533
F (ppm)	-	8477
Total	99.26	97.49

### Charge preparation

Depending on temperature, the experimental charges were made using either gold (≤ 1040 °C) or gold-palladium alloy (> 1040 °C). Sections of tubing 1.5 cm long and ~ 0.5 cm diameter were arc-welded at one end. They were first loaded with different amounts of water and then silver oxalate. The quantity of silver oxalate was calculated to retain a fixed fluid/silicate ratio. Approximately 30 mg of powdered rock was then added to the capsule, maintaining a fluid/silicate wt% ratio of about 0.1. The H_2_O/CO_2_ ratios (XH_2_O_in_= H_2_O/H_2_O+CO_2_ in moles), ranged from 1 down to nominally dry conditions (i.e. only CO_2_ added). Note that the vessel pressurising system uses H_2_, and this will lead to some water production in these runs, according to the equation Fe_2_O_3_+H_2_=FeO+H_2_O. The capsules were then welded shut and re-weighed. They were left for ~ 14 h in an oven at 100 °C to establish a homogeneous volatile distribution within the charge. Capsules were subsequently loaded to the vessel and annealed at HP-HT for several hours. After quenching and recovery, capsules showing weight differences (pre- and post-run) of less than 0.3 mg were considered successful.

### Experimental apparatus

A typical run consisted of a set of six capsules loaded with different XH_2_O_in_ and an extra one containing either CoPd or NiPd metallic sensors to record the prevailing oxygen fugacity (Taylor et al. 1992). Experiments were run in Internally Heated Pressure Vessels (IHPV) operating vertically, loaded with Ar–H_2_ mixtures at room temperature to achieve the desired *f*O_2_ conditions (QFM or Ni-NiO (NNO) equilibria, see below). Total pressure was recorded by a transducer calibrated against a Heise Bourdon gauge with an uncertainty of 2 MPa. Double-winding kanthal or Mo furnaces were used, ensuring near-isothermal conditions (gradient < 2–3 °C/cm) along a 3 cm long hot spot. Temperature was measured using S-type thermocouples with an accuracy of ± 5 °C. A drop-quench technique was used to terminate experiments, producing isobaric cooling rates of > 100 °C/s (Andújar et al. 2015, 2017; Scaillet et al. 2008). A successful drop quench was accompanied by a transient rise in total pressure indicating that the sample holder with the capsules has fallen into the cold (bottom) part of the vessel.

### Analysis of charges

Experimental charges were opened and mounted in epoxy resin for analysis. They were imaged using the FEI SEM at the University of Cambridge, and the H_2_O and CO_2_ contents of quenched glasses were measured by ion microprobe at the Edinburgh NERC Ion Microprobe Facility.

The samples were gold-coated and analysed for CO_2_ contents on the Cameca ims-1270 instrument and for H_2_O (H^+^) on the Cameca ims-4f at the EIMF. Also measured in the glass were Ti (to check for consistency across all measurements) and a selection of incompatible trace elements. On the ims-1270, a 3.8 nA primary beam at 22 keV was focussed to a spot size of 15–20 μm, using a 1-min pre-sputter with a 10 μm × 10 μm raster. The final eight of twenty cycles were retained. Secondary ions were collected at 10 kV with a 50 eV offset. The CO_2_ working curves were constructed from eight basaltic standards, with a compositional range from 0 to 2366 ppm CO_2_. Five standards were re-analysed after every 15–20 measurements. Measured CO_2_ and H_2_O contents are reported in Table 2.

**Table 2:** Experimental runs in this project, including CO_2_ and H_2_O as measured by SIMS

Run	*P* (MPa)	T (°C)	H_2_Omelt (wt%)	CO_2_ (ppm)	XH_2_O init	XH_2_O final	H_2_O (fl.)(g)	CO_2_ (fl.)(g)	XH_2_O (fl.)	fH_2_O	log fO_2_	ΔNNO	ΔQFM	fH_2_ (MPa)
Nab-11-A1	200	1000	5.50	Nd	1.00	1.00	1.3 × 10^-03^	0.0	1	1864	-11.5	-1.3	-0.6	5.37
Nab-11-A2	200	1000	5.00	Nd	0.92	0.88	9.5 × 10^-04^	6.2 × 10^-04^	0.79	1695	-11.6	-1.3	-0.7	
Nab-11-A3	200	1000	4.60	Nd	0.81	0.73	4.6 × 10^-04^	1.1 × 10^-03^	0.50	1559	-11.6	-1.3	-0.8	
Nab-11-A4	200	1000	4.30	Nd	0.74	0.68	Nd	Nd	Nd	1457	-11.7	-1.4	-0.8	
Nab-11-A5	200	1000	3.30	Nd	0.62	0.54	Nd	Nd	Nd	1118	-11.9	-1.6	-1.1	
Nab-11-A6	200	1000	Nd	Nd	0.00	0.00	0.0	0.0	0.0	186	-13.5	-3.2	-2.6	
Nab-11-B1	200	1040	4.74	4	1.00	1.00	1.5 × 10^-03^	0.0	1.00	1915	-10.6	-0.9	-0.3	4.16
Nab-11-B2	200	1040	4.05	391	0.95	0.90	1.2 × 10^-03^	3.3 × 10^-04^	0.90	1631	-10.7	-1.1	-0.5	
Nab-11-B3	200	1040	3.28	616	0.85	0.73	5.0 × 10^-04^	6.2 × 10^-04^	0.66	1318	-10.9	-1.3	-0.7	
Nab-11-B4	200	1040	2.71	850	0.73	0.66	7.6 × 10^-04^	1.4 × 10^-03^	0.57	1087	-11.1	-1.5	-0.8	
Nab-11-B5	200	1040	2.17	752	0.56	0.50	3.3 × 10^-04^	1.9 × 10^-03^	0.30	871	-11.3	-1.7	-1.0	
Nab-11-B6	200	1040	Nd	Nd	0.00	0.00	7.2 × 10^-05^	2.9 × 10^-03^	0.06	192	-12.6	-3.0	-1.0	
Nab-11-C1	100	1040	3.63	0	1.00	1.00	2.1 × 10^-03^	0.0	1.00	960	-11.1	-1.4	-0.8	3.51
Nab-11-C2	100	1040	3.38	83	0.92	0.89	1.4 × 10^-03^	5.5 × 10^-04^	0.86	893	-11.1	-1.5	-0.8	
Nab-11-C3	100	1040	3.22	169	0.79	0.73	9.6 × 10^-04^	1.3 × 10^-03^	0.64	850	-11.2	-1.5	-0.9	
Nab-11-C4	100	1040	3.15	262	0.71	0.64	4.1 × 10^-04^	1.5 × 10^-03^	0.40	831	-11.2	-1.6	-0.9	
Nab-11-C5	100	1040	2.23	326	0.59	0.53	4.0 × 10^-04^	2.0 × 10^-03^	0.33	588	-11.5	-1.9	-1.2	
Nab-11-C6	100	1040	Nd	Nd	0.00	0.00	6.8 × 10^-05^	2.9 × 10^-03^	0.05	96	-13.1	-3.4	-1.5	
Nab-11-D1	400	1100	5.74	223	1.00	1.00	1.2 × 10^-03^	0.0	1.00	4369	-9.5	-0.7	0.0	7.38
Nab-11-D2	400	1100	5.52	868	0.92	0.81	7.7 × 10^-04^	5.5 × 10^-04^	0.77	4176	-9.5	-0.7	-0.1	
Nab-11-D3	400	1100	5.12	1206	0.83	0.47	1.5 × 10^-04^	9.7 × 10^-04^	0.27	3864	-9.6	-0.8	-0.1	
Nab-11-D4	400	1100	4.43	1675	0.76	0.41	1.6 × 10^-04^	1.3 × 10^-03^	0.23	3332	-9.7	-0.9	-0.3	
Nab-11-D5	400	1100	3.94	1679	0.58	0.00	0.0	1.9 × 10^-03^	0.00	2963	-9.8	-1.0	-0.4	
Nab-11-D6	400	1100	2.66	2361	0.00	0.00	0.0	2.7 × 10^-03^	0.00	1991	-10.6	-1.8	-1.2	
Nab-11-E1	400	1040	5.80	0	1.00	1.00	1.3 × 10^-03^	0.0	1.00	4311	-10.1	-0.5	0.2	5.26
Nab-11-E2	400	1040	6.42	656	0.93	0.88	5.4 × 10^-04^	4.4 × 10^-04^	0.75	4750	-10.0	-0.4	0.3	
Nab-11-E3	400	1040	6.00	1054	0.84	0.72	3.1 × 10^-05^	8.6 × 10^-04^	0.08	4427	-10.1	-0.4	0.2	
Nab-11-E4	400	1040	4.94	1062	0.70	0.46	0.0	1.3 × 10^-03^	0.00	3644	-10.2	-0.6	0.0	
Nab-11-E5	400	1040	4.31	1469	0.58	0.40	0.0	2.0 × 10^-03^	0.00	3171	-10.4	-0.7	-0.1	
Nab-11-E6	400	1040	Nd	Nd	0.00	0.00	8.0 × 10^-05^	2.0 × 10^-03^	0.09	431	-12.1	-2.5	-0.5	
Nab-11-F1	100	1100	3.61	0	1.00	1.00	1.9 × 10^-03^	0.0	1.00	976	-9.8	-1.0	-0.3	2.23
Nab-11-F2	100	1100	3.46	43	0.95	0.92	1.5 × 10^-03^	3.5 × 10^-04^	0.91	935	-9.8	-0.9	-0.3	
Nab-11-F3	100	1100	3.25	137	0.86	0.74	7.9 × 10^-04^	7.8 × 10^-04^	0.71	877	-9.9	-0.9	-0.4	
Nab-11-F4	100	1100	3.05	199	0.78	0.67	8.4 × 10^-04^	1.2 × 10^-03^	0.63	823	-9.9	-1.0	-0.4	
Nab-11-F5	100	1100	2.75	280	0.63	0.42	3.6 × 10^-04^	1.8 × 10^-03^	0.32	741	-10.0	-1.1	-0.5	
Nab-11-F6	100	1100	2.09	Nd	0.00	0.00	0.0	3.2 × 10^-03^	0.00	565	-10.2	-1.3	-0.8	
Nab-11-G1	200	1100	4.76	0	1.00	1.00	1.1 × 10^-03^	0.0	1.00	1972	-9.1	-0.3	0.4	2.03
Nab-11-G2	200	1100	3.91	390	0.84	0.70	7.0 × 10^-04^	9.1 × 10^-04^	0.65	1614	-9.2	-0.3	0.2	
Nab-11-G3	200	1100	3.58	549	0.69	0.22	1.6 × 10^-05^	1.5 × 10^-03^	0.02	1476	-9.3	-0.4	0.2	
Nab-11-G4	200	1100	2.72	808	0.34	0.00	0.0	2.8 × 10^-03^	0.00	1119	-9.6	-0.7	-0.1	
Nab-11-G5	200	1100	1.90	972	0.00	0.00	0.0	3.3 × 10^-03^	0.00	781	-9.9	-1.0	-0.4	
Nab-11-H1	300	1075	5.35	3	1.00	1.00	1.6 × 10^-03^	0.0	1.00	3037	-9.7	-0.6	0.1	4.22
Nab-11-H2	300	1075	4.61	631	0.87	0.66	4.9 × 10^-04^	7.9 × 10^-04^	0.60	2604	-9.8	-0.6	0.0	
Nab-11-H3	300	1075	3.97	961	0.68	0.33	1.2 × 10^-04^	1.8 × 10^-03^	0.14	2237	-10.0	-0.7	-0.2	
Nab-11-H4	300	1075	3.23	1280	0.40	0.05	0.0	2.9 × 10^-03^	0.00	1816	-10.1	-0.9	-0.3	
Nab-11-H5	300	1075	2.70	1184	0.00	0.00	0.0	3.0 × 10^-03^	0.00	1519	-10.3	-1.1	-0.5	
Nab-11-I1	300	1100	5.74	0	1.00	1.00	1.2 × 10^-03^	0.0	1.00	3077	-9.0	-0.2	0.4	3.05
Nab-11-I2	300	1100	4.18	782	0.83	0.54	3.0 × 10^-04^	9.0 × 10^-04^	0.45	2228	-9.3	-0.4	0.2	
Nab-11-I3	300	1100	3.53	1106	0.63	0.31	1.3 × 10^-04^	1.8 × 10^-03^	0.14	1878	-9.5	-0.6	0.0	
Nab-11-I4	300	1100	2.89	1361	0.34	0.00	0.0	2.3 × 10^-03^	0.00	1534	-9.6	-0.8	-0.2	
Nab-11-I5	300	1100	1.84	1542	0.00	0.00	0.0	3.1 × 10^-03^	0.00	976	-10.0	-1.1	-0.6	
Nab-11-J1	100	1040	3.52	0.00	1.00	1.00	1.7 × 10^-03^	0.0	1.00	960	-9.5	0.1	0.7	0.587
Nab-11-J2	100	1040	3.13	169	0.83	0.75	1.1 × 10^-03^	1.0 × 10^-03^	0.72	852	-9.6	0.0	0.7	
Nab-11-J3	100	1040	2.69	295	0.65	0.44	2.9 × 10^-04^	1.7 × 10^-03^	0.30	731	-9.7	-0.1	0.5	
Nab-11-J4	100	1040	2.05	378	0.40	0.22	0.0	2.6 × 10^-03^	0.00	557	-10.0	-0.3	0.3	
Nab-11-J5	100	1040	1.49	270	0.00	0.00	0.0	3.0 × 10^-03^	0.00	405	-10.2	-0.6	0.0	

Uncertainties are ~ 0.1 wt% for H_2_O and 50 ppm for CO_2_

On the Cameca ims-4f, a 15 μm 5–6 nA primary beam was used with 15 keV energy, using a 1-min pre-sputter at 10 µm^2^. Secondary ions were collected at 4.5 kV with a 75 eV offset and using a field aperture to restrict the zone of collection and reduce noise for 1 H+. The trace elements (Sr, Zr, La, Ba, Ce, Th, U) were collected across the full field on glasses and selected plagioclase phenocrysts from natural samples, and peak-stripping was applied to remove influences from light REE oxides. The data were normalised to ^30^Si and then corrected using EPMA data.

The chemical composition of phases was measured at the University of Cambridge using a Cameca SX100 electron microprobe. Major elements were counted for 30–60 s and volatiles (S, Cl, F) for 60–90 s. Minerals were analysed using a 15 kV, 20 nA focussed 1 μm beam. Glass was analysed using a 15 kV, 6 nA beam, which was defocussed at 10 μm, always measuring Na first to limit Na-loss. Standards were analysed at regular intervals throughout the measurement period.

Modal analysis of the experimental phases and thin sections was made using the Scanning Electron Microscope (SEM) images and ImageJ software (Abràmoff et al. 2004), with oxides further analysed using electron backscatter diffraction (EBSD) on the FEI SEM in the University of Cambridge. Phase proportions were also calculated using mass balance, yielding comparable results.

### Experimental conditions

Following Donovan et al. (2018), the magma composition was investigated from 1000 to 1100 °C at ~ QFM, in the pressure range 100 to 400 MPa. One run was replicated at ~ NNO to explore the effect of *f*O_2_ on phase compositions. The *f*H_2_ of the experiments was determined using CoPd and NiPd metallic sensors (Taylor et al. 1992). The *f*O_2_ of any charge was determined using the dissociation constant of water (Robie et al. 1979), knowing the *f*H_2_O at the experimental temperature and pressure. For each capsule, the *f*H_2_O was calculated as the product of XH_2_O_fin_ and *f*H2O^o^, where *f*H_2_O^o^ is the fugacity of pure water (Burnham et al. 1969) and XH_2_O_fin_ is the final composition of the excess fluid. The latter quantity was determined by mass balance, knowing the amounts of CO_2_ and H_2_O dissolved in the quenched glass, as determined by ion probe, and subtracting those quantities from the amounts of H_2_O and CO_2_ loaded to the charge. As at fixed T, P, *f*H_2_, *f*O_2_ decreases with XH_2_O (e.g., Webster et al., 1987), in a single experimental series, the decrease in XH_2_O_fin_ from 1 to < 0.1 decreases *f*O_2_ by up to 2 log units below the QFM buffer (or 0.7 log units below NNO; Table 2).

The aim here was to approach the equilibrium conditions that the magma achieved at the final stage before eruption, as represented by microlites and phenocryst rims. As a result, the duration of the experiments was 24 h, consistent with other studies of basaltic compositions. The euhedral shape of experimental crystals and the homogeneous distribution of phases in the experimental samples also suggests that experiments were close to equilibrium.

## Results

The experimental data were assessed for Fe-loss using mass balance calculations. Some of the higher temperature experiments (done in AuPd capsules) had experienced some degree of Fe-loss. This was most pronounced at high temperatures and low pressures (F series experiments), where Fe-loss was up to 25% (in charge F6). These charges are omitted from modelling, below. Electron microprobe analyses were also assessed, and some Na-loss had occurred in the first set of measurements of the A-series experiments, but not in the second set of analyses, which are reported here.

**Fig. 3:**
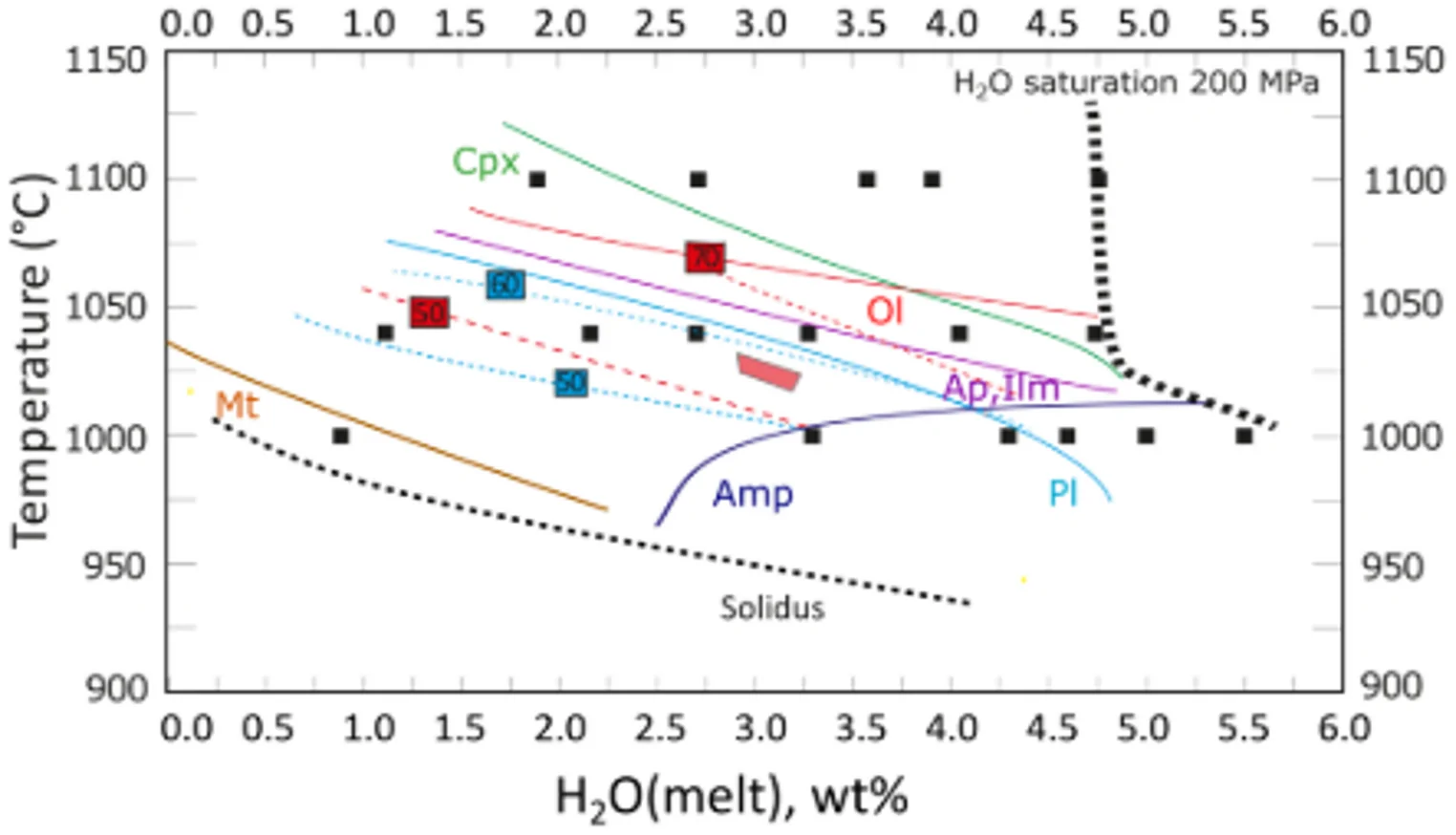
Phase equilibria as a function of H_2_O_melt_ and temperature at 200 MPa. Black symbols are experimental charges. Phase labels lie inside their stability field. Red dashed lines give the Fo content of Ol. Blue dashed lines give the An content of Pl. Black medium dotted line gives the approximate position of the solidus. Black thick dotted curve corresponds to the H_2_O-saturation curve at 200 MPa. The red polygon indicates the pre-eruptive conditions as inferred from phenocryst rim compositions and phenocryst proportion in the rock. Water content of nominally “dry” charges could not be measured and is estimated from the XH_2_O_fin_ and SIMS measurements of the other charges in the run

**Fig. 4:**
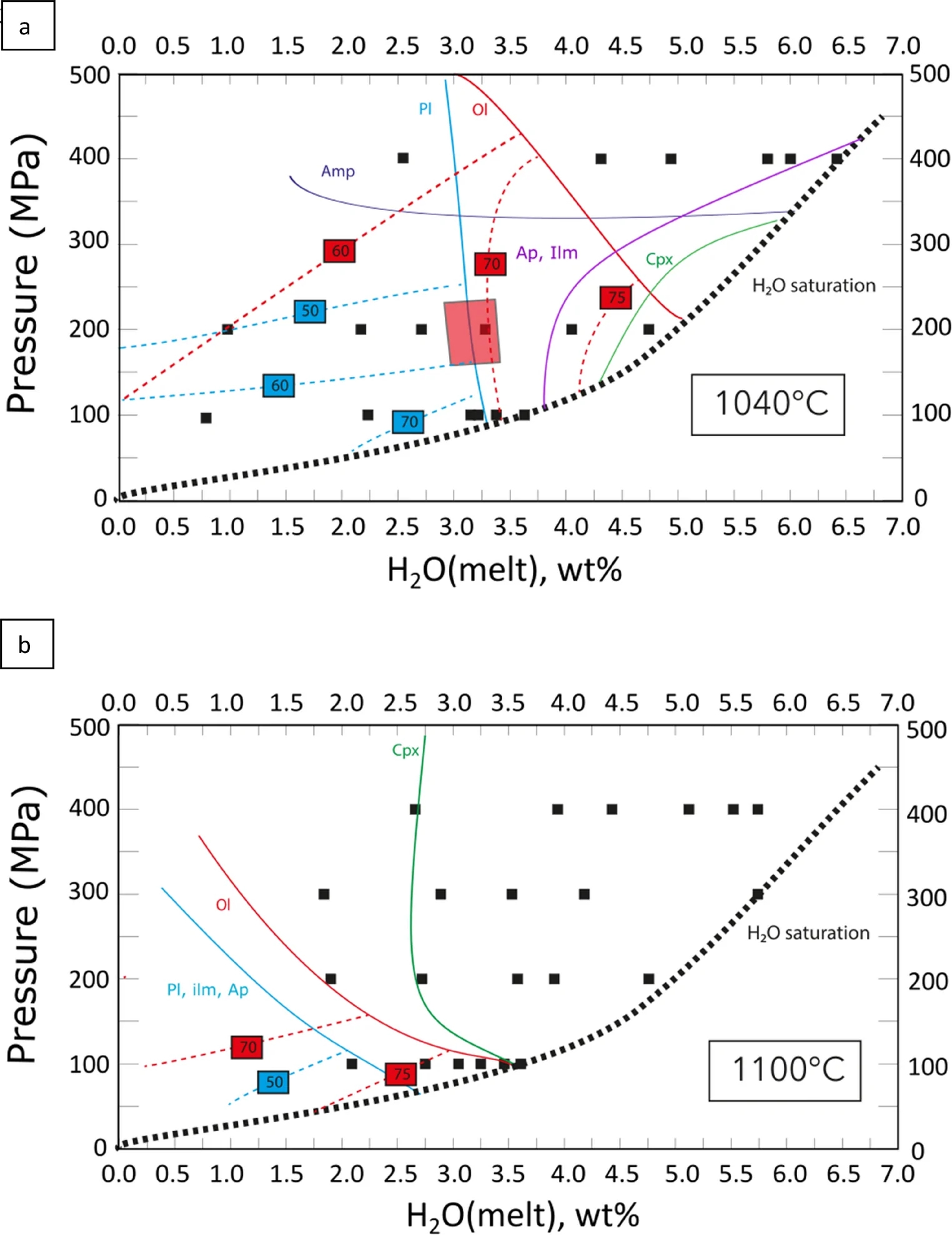
**a** Phase equilibria as a function of H_2_O_melt_ and pressure at **a** 1040 °C and **b** 1100 °C. Black squares are experimental charges. Phases labelling the different curves lie inside their stability field. Red dashed lines give the Fo content of Ol. Blue dashed lines give the An content of Pl. Black thin dashed lines give the crystal fraction (wt%). Black thick dotted curve corresponds to the H_2_O-saturation curve. The red rectangle indicates the pre-eruptive conditions as inferred from phenocryst rim compositions and phenocryst proportion in the rock. Mt is not shown but was present in the 100 MPa experiment at ~NNO and not at ~QFM

Experimental mineral phases identified in the run products are Ol, Cpx, Pl, Amp, Ilm, Mt and Ap. The experimental results are reported in Table 3; the stability of the different phases and their dependence with experimental P–T–H_2_O_melt_ are shown in a series of polybaric-polythermal or isobaric-isothermal sections (Figs. 3 and 4).

We first discuss the data obtained at 200 MPa (Fig. 3). Cpx is the liquidus phase in the dry part of the diagram, replaced by Ol in the H_2_O-rich region (> 4.5 wt%). The next phases to crystallise are Ilm and Ap, both having curves parallel to that of Ol. Pl appears at T∼1000 °C when H_2_O_melt_ = 4.3 wt% (XH_2_O = 0.68), also with a negative slope in this projection. Amp is stable at T < 1040 °C and occupies the H_2_O-rich region of the diagram (H_2_O_melt_ >3 wt% or XH_2_O_fin_ of 1 to 0.5). Mt appears only in H_2_O-poor charges below 1040 °C. It is also produced under wetter conditions if *f*O_2_ is closer to NNO. Isopleths of An and Fo contents in Pl and Ol show that both phases become more mafic at high H_2_O_melt_. The data show that at 200 MPa, Pl with > An_60_ is unlikely. Similarly, Ol with > Fo_75_ would require H_2_O-saturated conditions.

The 1040 °C-polybaric section shows that Ol is the liquidus phase at 200 MPa (Fig. 4a). The slope of its curve is not well constrained by our data but appears to be negative in this projection. Pl displays a negatively but steeply sloped in-curve. In contrast, the Cpx curve has a positive slope, and is the liquidus phase above 200 MPa. Mt (run J) has a narrow stability field which will be expanded towards higher H_2_O _melt_ and pressure by higher *f*O_2_. The orientation of An isopleths shows that the crystallisation of An-rich Pl requires low pressure. Production of Pl with > An_70_ is unlikely with this bulk magma composition at this temperature. The isopleths for Fo are steeper, in particular in the H_2_O-rich part of the stability field of Ol. Fo-rich Ol requires lower pressure to crystallise. Magmas containing more than 1 wt% H_2_O_melt_ will essentially melt upon isothermal decompression in the pressure interval 100–400 MPa. The increase in crystallinity with pressure in the H_2_O-rich part of the section is due to the profuse crystallisation of Amp at mid-crustal pressure. There is thus a crystal poor-window at 200–300 MPa and H_2_O-rich conditions.

The 1100 °C-polybaric section (Fig. 4b) shows that increasing temperature shifts all stability fields toward lower H_2_O_melt_. In addition, neither Amp nor Mt are present. Ol is the first to crystallise at 100 MPa, followed by Cpx and Pl. At this temperature, the near liquidus co-precipitation of Ol, Cpx and Pl requires pressures below 150 MPa and H_2_O_melt_<2wt% (or even around 100 MPa if Ap is taken into consideration). Similarly, to crystallise Fo > 75 Ol requires a pressure of about 100 MPa and H_2_O_melt_=3 wt%. Although less constrained than in the polybaric section at 1040 °C, crystallisation of An-rich Pl would also require low pressures, and generation of compositions more calcic than An_60_ is unlikely from this bulk magma composition at this temperature. The different isopleths of crystal proportion are negatively sloped in this projection, showing that a decompressing magma at a constant temperature with less than 3 wt% H_2_O_melt_ will crystallise.

**Table 3:** Phase assemblages for the experimental runs, including modal analysis of charges and measurements of H2O (wt%) by ion probe

Run	XH_2_O init	H_2_Owt% melt	X H_2_O_FIN_	Phase assemblage	ol	cpx	ox	pl	amp	ap	glass	xtals	
**200 MPa**,** 1000 °C**,** 6.1 pH**_**2**_													
Nab-11-A1	1.00	5.50	1.00	ol+cpx+ilm+amp+ap+liq	2.21	7.74	1.30	0.00	21.24	1.11	66.97	33.03	
Nab-11-A2	0.92	5.00	0.88	ol+cpx+ilm+amp+ap+liq	2.18	8.74	1.69	0.00	22.75	1.09	63.50	36.50	
Nab-11-A3	0.81	4.60	0.73	ol+cpx+ilm+amp+ap+liq	3.35	8.93	1.41	0.00	24.01	1.12	61.12	38.88	
Nab-11-A4	0.74	4.30	0.68	ol+cpx+ilm+amp+plg+ap+liq	4.70	10.56	2.78	4.12	24.76	1.17	52.53	47.47	
Nab-11-A5	0.62	3.30	0.54	ol+cpx+ilm+amp+plg+ap+liq	5.90	12.06	1.10	5.88	27.19	nd	47.87	52.13	
Nab-11-A6	0.00	nd	0.00	ol+cpx+ilm+plg+mt+ap+liq	nd	nd	8.00	43.70	0.00	nd	2.00	98.00	
**200 MPa**,** 1040 °C**,** 6.1 pH**_**2**_													
Nab-11-B1	1.00	4.74	1.00	ol+liq	0.05	0.00	0.00	0.00	0.00	0.00	100.00	0.00	
Nab-11-B2	0.95	4.05	0.90	ol+cpx+liq	0.23	0.40	0.00	0.00	0.00	0.00	99.00	1.00	
Nab-11-B3	0.85	3.28	0.73	ol+cpx+ilm+ap+liq	2.04	8.16	2.04	0.00	0.00	nd	85.67	14.33	
Nab-11-B4	0.73	2.71	0.66	ol+cpx+ilm+plg+ap+liq	4.30	13.96	1.32	7.24	0.00	nd	73.18	26.82	
Nab-11-B5	0.56	2.17	0.50	ol+cpx+ilm+plg+ap+liq	8.41	19.79	1.93	22.88	0.00	nd	47.00	53.00	
Nab-11-B6	0.00	nd	0.06	ol+cpx+ilm+plg+ap+liq	8.57	17.86	0.37	39.87	0.00	nd	33.34	66.66	
**100 MPa**,** 1040 °C**,** 6 pH**_**2**_													
Nab-11-C1	1.00	3.63	1.00	ol+cpx+ilm+ap+liq	2.11	8.98	0.85	0.00	0.00	nd	88.06	11.94	
Nab-11-C2	0.92	3.38	0.89	ol+cpx+ilm+ap+liq	9.29	22.98	1.02	0.00	0.00	nd	66.72	33.28	
Nab-11-C3	0.79	3.22	0.73	ol+cpx+ilm+plg+ap+liq	9.61	22.92	1.48	5.20	0.00	nd	60.79	39.21	
Nab-11-C4	0.71	3.15	0.64	ol+cpx+ilm+plg+ap+liq	11.37	24.73	1.68	16.68	0.00	nd	45.54	54.46	
Nab-11-C5	0.59	2.23	0.53	ol+cpx+ilm+plg+ap+liq	14.11	24.05	2.14	16.05	0.00	nd	43.65	56.35	
Nab-11-C6	0.00	nd	0.05	ol+cpx+ilm+plg+ap+liq	nd	nd	nd	nd	0.00	nd	nd	100.00	
**400 MPa**,** 1100 °C**,** 6 pH**_**2**_													
Nab-11-D1	1.00	5.74	1.00	Liq	0.00	0.00	0.00	0.00	0.00	0.00	100.00	0.00	
Nab-11-D2	0.92	5.52	0.81	Liq	0.00	0.00	0.00	0.00	0.00	0.00	100.00	0.00	
Nab-11-D3	0.83	5.12	0.46	Liq	0.00	0.00	0.00	0.00	0.00	0.00	100.00	0.00	
Nab-11-D4	0.76	4.43	0.40	Liq	0.00	0.00	0.00	0.00	0.00	0.00	100.00	0.00	
Nab-11-D5	0.58	3.94	0.00	Liq	0.00	0.00	0.00	0.00	0.00	0.00	100.00	0.00	
Nab-11-D6	0.00	2.66	0.00	cpx+liq	0.00	10.49	0.00	0.00	0.00	0.00	89.81	10.39	
**400 MPa**,** 1040 °C**,** 6 pH**_**2**_													
Nab-11-E1	1.00	5.80	1.00	cpx+ilm+amp+liq	0.00	15.10	0.41	0.00	10.74	0.00	73.76	26.24	
Nab-11-E2	0.93	6.42	0.88	cpx+ilm+amp+ap+liq	0.00	10.57	1.29	0.00	18.35	0.81	68.98	31.02	
Nab-11-E3	0.84	6.00	0.72	cpx+ilm+amp+ap+liq	0.00	9.53	1.10	0.00	23.87	1.30	64.20	35.80	
Nab-11-E4	0.70	4.94	0.46	cpx+ilm+amp+ap+liq	0.00	20.77	0.97	0.00	20.87	1.48	55.91	44.09	
Nab-11-E5	0.58	4.31	0.40	cpx+ilm+amp+ap+liq	0.00	24.65	0.74	0.00	17.99	1.21	55.42	44.58	
Nab-11-E6	0.00	nd	0.09	ol+cpx+ilm+amp+plg+ap+liq	5.48	10.08	2.37	nd	17.30	nd	40.71	59.29	
**100 MPa**,** 1100 °C**,** 6 pH**_**2**_													
Nab-11-F1	1.00	3.61	1.00	Liq	0.00	0.00	0.00	0.00	0.00	0.00	100.00	0.00	
Nab-11-F2	0.95	3.46	0.92	ol+cpx+liq	1.01	nd	0.00	0.00	0.00	0.00	98.99	1.01	
Nab-11-F3	0.86	3.25	0.74	ol+cpx+liq	1.01	1.01	0.00	0.00	0.00	0.00	97.99	2.01	
Nab-11-F4	0.78	3.05	0.67	ol+cpx+liq	1.01	1.51	0.00	0.00	0.00	0.00	95.98	4.02	
Nab-11-F5	0.63	2.75	0.42	ol+cpx+liq	1.01	1.52	0.00	0.00	0.00	0.00	95.96	4.04	
Nab-11-F6	0.00	2.09	0.00	ol+cpx+ilm+plg+ap+liq	6.26	7.29	2.28	15.31	0.00	nd	71.63	28.37	
**200 MPa**,** 1100 °C**,** 6.2 pH**_**2**_													
Nab-11-G1	1.00	4.76	1.00	Liq	0.00	0.00	0.00	0.00	0.00	0.00	100.00	0.00	
Nab-11-G2	0.84	3.91	0.69	Liq	0.00	0.00	0.00	0.00	0.00	0.00	100.00	0.00	
Nab-11-G3	0.69	3.58	0.22	Liq	0.00	0.00	0.00	0.00	0.00	0.00	100.00	0.00	
Nab-11-G4	0.34	2.72	0.00	Liq	0.00	0.00	0.00	0.00	0.00	0.00	100.00	0.00	
Nab-11-G5	0.00	1.90	0.00	cpx+liq	0.00	2.00	0.00	0.00	0.00	0.00	98.00	2.00	
**300 MPa**,** 1075 °C**,** 6 pH**_**2**_													
Nab-11-H1	1.00	5.35	1.00	Liq	0.00	0.00	0.00	0.00	0.00	0.00	100.00	0.00	
Nab-11-H2	0.87	4.61	0.66	Liq	0.00	0.00	0.00	0.00	0.00	0.00	100.00	0.00	
Nab-11-H3	0.68	3.97	0.33	Liq	0.00	0.00	0.00	0.00	0.00	0.00	100.00	0.00	
Nab-11-H4	0.40	3.23	0.05	cpx+liq	0.00	17.06	0.00	0.00	0.00	0.00	82.94	17.06	
Nab-11-H5	0.00	2.70	0.00	cpx+liq	0.00	33.32	0.00	0.00	0.00	0.00	66.68	33.32	
**300 MPa**,** 1100 °C**,** 6 pH**_**2**_													
Nab-11-I1	1.00	5.74	1.00	Liq	0.00	0.00	0.00	0.00	0.00	0.00	100.00	0.00	
Nab-11-I2	0.83	4.18	0.54	Liq	0.00	0.00	0.00	0.00	0.00	0.00	100.00	0.00	
Nab-11-I3	0.63	3.53	0.31	Liq	0.00	0.00	0.00	0.00	0.00	0.00	100.00	0.00	
Nab-11-I4	0.34	2.89	0.00	Liq	0.00	0.00	0.00	0.00	0.00	0.00	100.00	0.00	
Nab-11-I5	0.00	1.84	0.00	cpx+liq	0.00	1.00	0.00	0.00	0.00	0.00	99.00	1.00	
**100 MPa**,** 1040 °C**,** 2.7 pH**_**2**_													
Nab-11-J1	1.00	3.52	1.00	cpx+ilm+mt+liq	0.00	nd	nd	0.00	0.00	0.00	98.00	2.00	
Nab-11-J2	0.83	3.13	0.75	ol+cpx+ilm+mt+liq	6.96	nd	0.04	0.00	0.00	0.00	90.78	9.22	
Nab-11-J3	0.65	2.69	0.44	ol+cpx+ilm+mt+liq	14.34	nd	0.02	0.00	0.00	0.00	85.64	14.36	
Nab-11-J4	0.40	2.05	0.22	ol+cpx+ilm+plg+mt+liq	18.46	4.12	0.78	6.72	0.00	0.00	69.93	30.07	
Nab-11-J5	0.00	1.49	0.00	ol+cpx+ilm+plg+mt+liq	21.49	5.37	1.80	21.90	0.00	0.00	52.81	47.19	

“nd” denotes that the phase was present, but too small to be included in the modal analysis, or that there were no patches of glass large enough to be measured for H_2_O content by ion probe. Ilmenite and magnetite were only found together in two experiments (J series, at NNO, and charge A6) and were modally counted together as oxides in the “oxide” column; they were too small to distinguish individually for modal analysis

We further regressed the data for melt wt%, T, P and H_2_O, and fitted them to the following equation:

1 $$\begin{aligned} {\mathrm{F}}\_{\mathrm{liq}}\left( {{\mathrm{wt}}\% } \right)\,= & \,100{\text{ }}/{\text{ }}(1\,+\,\exp ( - ({\mathrm{T}}\left( {^\circ {\mathrm{C}}} \right){\text{ }} - {\text{ }}(1039.63\left( { \pm \,9.0814} \right)\, \\ & +\,0.01464\left( { \pm \,0.003304} \right) \times {\mathrm{P}}\left( {{\mathrm{bar}}} \right){\text{ }} - 15.79\left( { \pm \,2.7090} \right) \\ & \times {H_2}O\left( {{\mathrm{wt}}\% } \right)))/25.88\left( { \pm \,3.8917} \right))){\text{ }}\left( {{\mathrm{RMSE}}\,=\,11.08} \right) \\ \end{aligned} $$

**Fig. 5:**
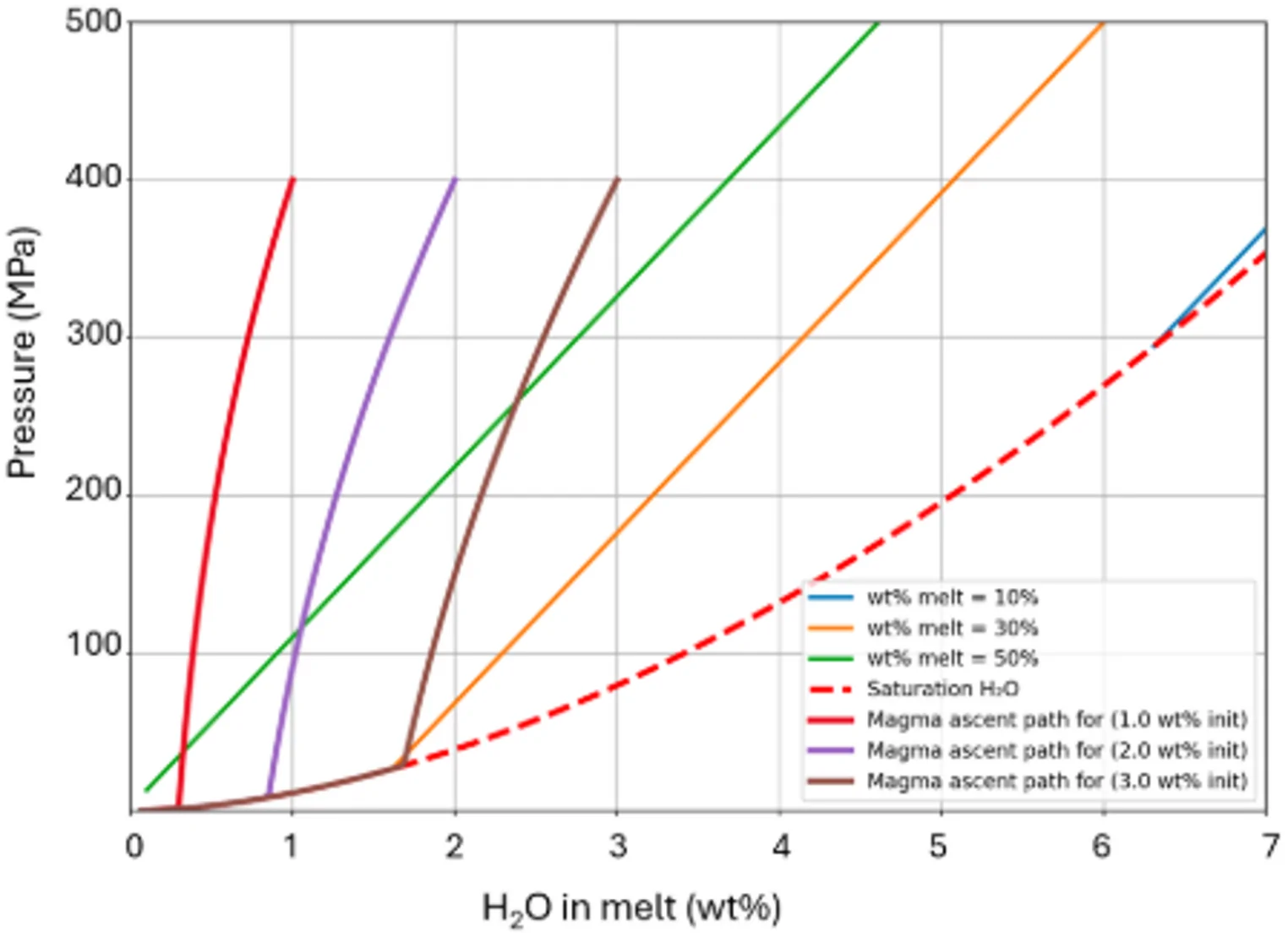
Model showing the behaviour of magmas of different melt fractions for a range of pressures and water contents, based on Eq. (1)

This equation has been used in Fig. 5 to show isopleths of constant melt fraction (10, 30, 50 wt%), the H_2_O saturation curve (from Lesne et al., 2011) and the paths followed by a decompressing magma with different initial amounts of H_2_O dissolved at 400 MPa (1, 2 and 3 wt% H_2_O_melt_), calculated assuming a bulk H_2_O content and a constant temperature of 1040 °C. The curves show that in all three cases, the magma melts upon decompression (addition of CO_2_ to the system will drive the calculated paths toward drier conditions owing to the fact that some dissolved H_2_O will be transferred to the excess gas). This effect will be, however, counteracted by cooling during ascent as is discussed below in the discussion section.

### Phase proportions

The crystal content in the charges varies from 0 wt% to 100 wt% (Table 3). At a given pressure, the amount of crystallization increases with decreasing H_2_O_melt_ and T, as expected. The proportion of Cpx reaches 20 wt% at low temperatures, similar to that of Amp (20 wt%) when present. Pl reaches up to 40 wt% at 1000 °C, being the dominant phase in crystal-rich charges. The proportion of Ol seems to be sensitive to P, since at pressures below 200 MPa, its amount is < 9 wt%, while at 100 MPa it reaches 14 wt%.

**Fig. 6:**
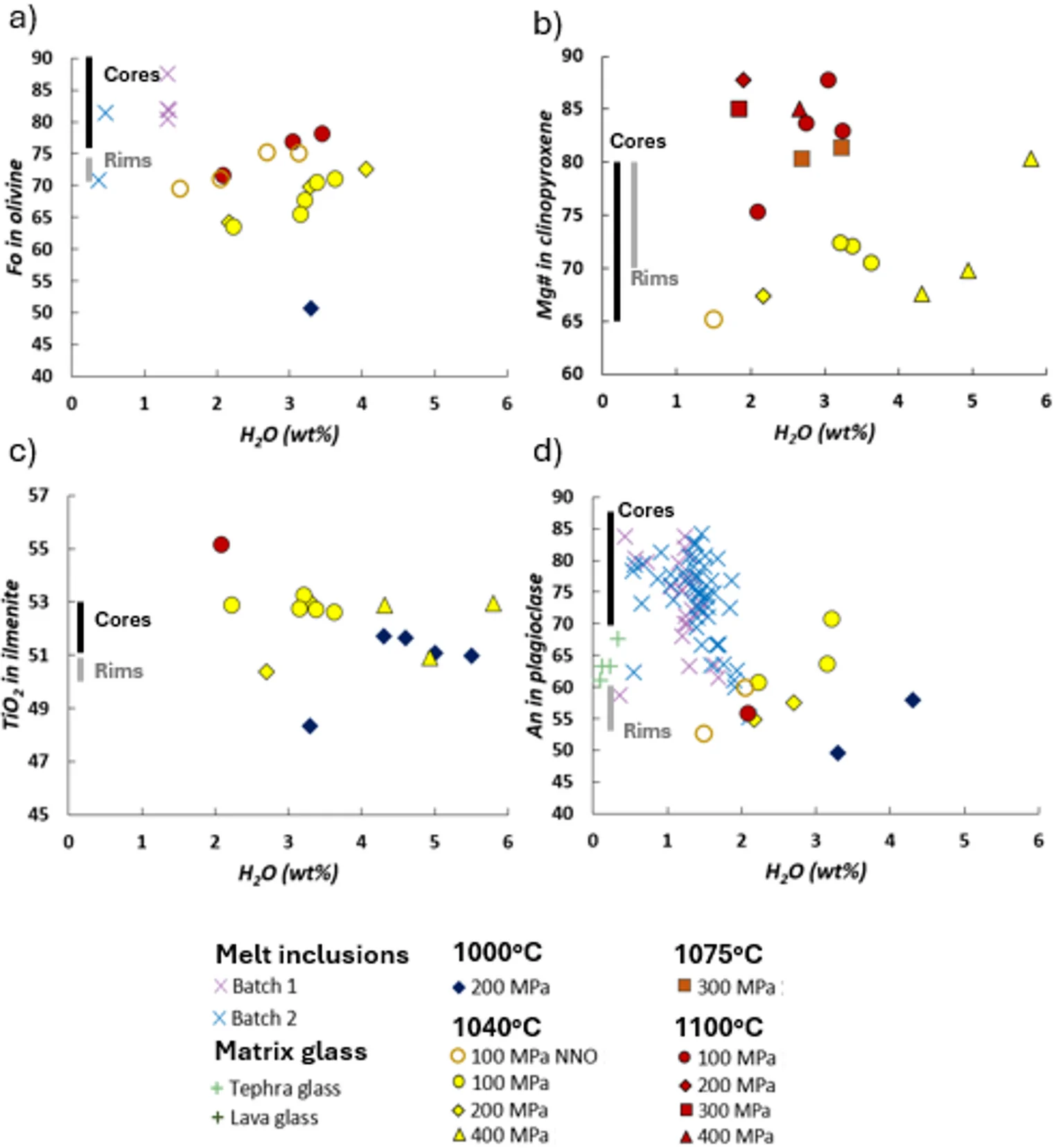
Relationships between melt water content and mineral compositions for experimental runs and melt inclusion data. “Rims” refers to the approximate compositions of the phenocryst rims and microlites in natural samples represented by the grey lines, taken to represent the last equilibration stage (in relation to the y axis only); core compositions are indicated by black lines. Water contents from melt inclusions (batches 1 and 2, X symbols) and matrix glass (+ symbols) are also shown (Donovan et al. 2018), with the compositions of phenocryst cores/rims in equilibrium with them. Open symbols are at NNO; closed are at QFM. Circles are experiments at 100 MPa; diamonds are 200 MPa; squares are 300 MPa and triangles 400 MPa

### Phase compositions

The Fo content of Ol increases with increasing H_2_O_melt_, *f*O_2_ and temperature (Fig. 6a), varying between Fo_78_ and Fo_63_. For instance, at 100 MPa an increase of 2 wt% H_2_O_melt_ increases Fo by 6–7 mol%, while a temperature increase of 50–60 °C increases Fo by about 10 mol% at fixed H_2_O_melt_. The CaO content is highest (up to 1.2 wt%) in the dry charges at lower temperatures. Linear regression of the QFM data in terms of Fo content vs. temperature and H_2_O_melt_ gives the following equation:

2 $$\begin{aligned} {\text{Fo }}= & {\text{ }}(0.00{\mathrm{2}}( \pm \,0.0{\mathrm{8}}) \times {\mathrm{T}}({\mathrm{C}}^\circ )\,+\,{\mathrm{2}}.{\mathrm{71}}( \pm \,0.{\mathrm{87}})) \\ & \times {{\mathrm{H}}_{\mathrm{2}}}{{\mathrm{O}}_{{\mathrm{melt}}}}\,+\,0.{\mathrm{14}}( \pm \,0.{\mathrm{25}}) \times {\mathrm{T}}(^\circ {\mathrm{C}})\, - \,{\mathrm{92}}( \pm \,{\mathrm{263}}) \\ \end{aligned} $$

Or, rearranging for T,

3 $$\begin{aligned} {\mathrm{T}}(^\circ {\mathrm{C}}\, \pm \,{\mathrm{6}}){\text{ }}= & {\text{ }}({\mathrm{Fo}}\,+\,{\mathrm{92}}( \pm \,{\mathrm{263}}){\text{ }} - {\text{ }}({\mathrm{2}}.{\mathrm{71}}( \pm \,0.{\mathrm{87}}) \times {{\mathrm{H}}_{\mathrm{2}}}{{\mathrm{O}}_{{\mathrm{melt}}}})){\text{ }}/{\text{ }} \\ & (0.00{\mathrm{2}}( \pm \,0.0{\mathrm{8}}) \times {{\mathrm{H}}_{\mathrm{2}}}{{\mathrm{O}}_{{\mathrm{melt}}}}\,+\,0.{\mathrm{14}}( \pm \,0.{\mathrm{25}})) \\ \end{aligned} $$

This equation allows us to calculate temperature if H_2_O_melt_ and Fo contents are known. We stress that these calibrations (and those for Pl/melt below) are specific to the bulk composition used in the experiments and should not be used on other rocks.

Mg# in Cpx broadly increases with increasing temperature and increasing pressure (Fig. 6b), but, unlike Ol and Pl, the data show considerable spread which precludes derivation of an empirical formula relating experimental variables.

Amp is stable in two sets of experiments—at high pressure and low temperature (1040 °C, 400 MPa, and 1000 °C, 200 MPa). It does not however occur in the natural samples from 2011. A-site alkalis decrease with decreasing water content.

The TiO_2_ content of Ilm increases with increasing temperature (Fig. 6c) and decreases with increasing pressure and H_2_O_melt_. Mt crystals were too small to be analysed reliably.

As for Ol, Pl composition depends on P, T and H_2_O_melt_. The most calcic Pl (An_71_) crystallized at 1040 °C–100 MPa and 3.5 wt% H_2_O_melt_ whereas the least calcic (An_48_) occurs in charges held at 1040–1000 °C, 200 MPa with 3.5 wt% H_2_O_melt_ (Fig. 6d). Our data show that Pl compositional variation conforms to observations made in other experimental studies (e.g., Andujar et al. 2016). In general, at any fixed T, An-rich Pl requires low pressure and elevated H_2_O_melt_. Our data set can be fitted linearly to the following equation:

4 $$\begin{aligned} {\text{An }}= & {\text{ }}({\mathrm{T}}\left( {^\circ {\mathrm{C}}} \right){\text{ }}-{\text{ 985}}.{\mathrm{7}}\left( { \pm \,{\mathrm{17}}} \right)\, +\,{\mathrm{14}}.{\mathrm{67}} \\ &\left( { \pm \,{\mathrm{1}}.{\mathrm{99}}} \right)\times {{\mathrm{H}}_{\mathrm{2}}}{{\mathrm{O}}_{{\mathrm{melt}}}})/{\mathrm{1}}.{\mathrm{49}} \end{aligned}$$

Or, rearranging for T,

5 $$\begin{aligned} {\mathrm{T}}(^\circ {\mathrm{C}}\, \pm \,{\mathrm{5}})\,=&\,{\mathrm{985}}.{\mathrm{7}}\left( { \pm \,{\mathrm{17}}} \right)\,+\,{\mathrm{1}}.{\mathrm{49}}\left( { \pm \,0.{\mathrm{28}}} \right) \times \\ & {\mathrm{An}} - {\mathrm{14}}.{\mathrm{67}}\left( { \pm \,{\mathrm{1}}.{\mathrm{99}}} \right) \times {{\mathrm{H}}_{\mathrm{2}}}{{\mathrm{O}}_{{\mathrm{melt}}}} \end{aligned}$$

Compared to the equivalent experiment carried out at QFM, that done at ~ NNO (J series of experiments) produced a Fo-richer Ol, the NNO–1040 °C trend falling onto the 1100 °C–QFM one (Fig. 6a). This indicates that the use of Eq. 3 (calibrated from QFM data) on an Ol from this magma at NNO, would underestimate temperature by about 60 °C. In contrast Pl does not show any appreciable change (An_50–60_). Cpx and oxides were too small to be adequately analysed.

### Composition of experimental glasses

**Fig. 7:**
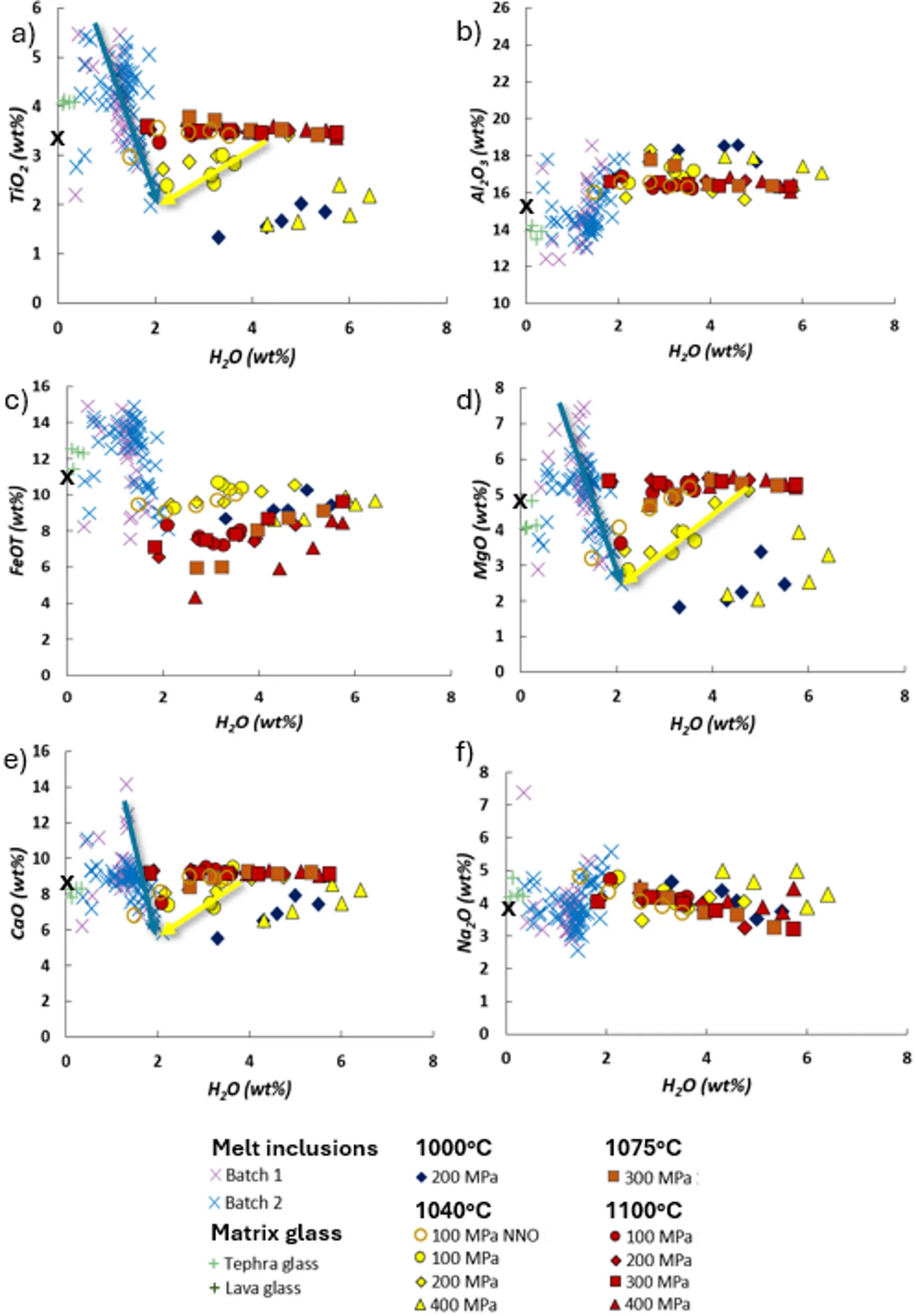
Compositions of experimental glasses, compared to glass from matrix and inclusions (from Donovan et al. 2018; + signs and x signs) The yellow arrow shows the effect of decreasing H_2_O_melt_ at 1040 °C, while the blue one corresponds to the trend displayed by natural MI. The “X” on the y axis is the starting composition for the charges

The experimental glass compositions are compared with the melt inclusions and matrix glasses measured by Donovan et al. (2018) in Fig. 7. The experimental glasses display smooth variations with experimental variables, reflecting the effects of crystallising minerals. For instance, TiO_2_ is lower in charges containing amphibole (which has 4–5 wt% TiO_2_) or when Ilm occurs in abundance. Similarly, charges lacking Pl display higher Al_2_O_3_ contents, while those crystallising abundant Cpx show a depletion (relative to the bulk rock) in CaO. FeO content is affected by Fe–Mg minerals but also to some extent by Fe-loss toward the capsules, in particular in charges made with with AuPd capsules at T > 1040 °C.

The variation of melt composition with P, T and H_2_O_melt_ can be used to calibrate a melt thermometer specific to Nabro melt compositions. In doing so we use a simple empirical equation such as that derived by Sisson and Grove (1993) for calc-alkaline basaltic melts, which has proved suitable for constraining the temperature of magma evolution via the analyses of MI, for use with our own dataset. We use glass compositions from charges saturated in at least one of the main phases (Ol, Pl, Cpx) and not affected by substantial Fe-loss, which corresponds to a total of 31 charges. The empirical equation is as follows:

6 $$\begin{aligned} {\mathrm{T}}^\circ {\text{C }} = & {\text{ 988}}.{\mathrm{33}}\left( { \pm {\mathrm{18}}.{\mathrm{9}}} \right){\text{ }} - {\text{ 9}}.{\mathrm{65}}\left( { \pm {\mathrm{2}}.{\mathrm{92}}} \right){\text{ }} \times {\text{ H}}_{{\mathrm{2}}} {\mathrm{O}}_{{{\mathrm{melt}}}} \left( {{\mathrm{wt}}\% } \right){\text{ }} \\ & + {\text{ }}0.00{\mathrm{977}}\left( { \pm 0.00{\mathrm{3}}0{\mathrm{8}}} \right){\text{ }} \times {\text{ }}\left( {{\mathrm{P}} - {\mathrm{1}}} \right){\text{ }} \\ & + {\text{ 163}}.{\mathrm{24}}\left( { \pm {\mathrm{32}}.{\mathrm{9}}} \right){\text{ }} \times {\text{ Mg}}\# \\ \end{aligned} $$

where H_2_O is in wt%, Mg# is the molar ratio MgO/(FeO + MgO) and P is in bar. This equation retrieves most experimental temperatures to within ± 13.7 °C (see supplementary Figure A9). Note that a variation of ± 1 wt% H_2_O_melt_ corresponds to a variation of ± 10 °C.

**Fig. 8:**
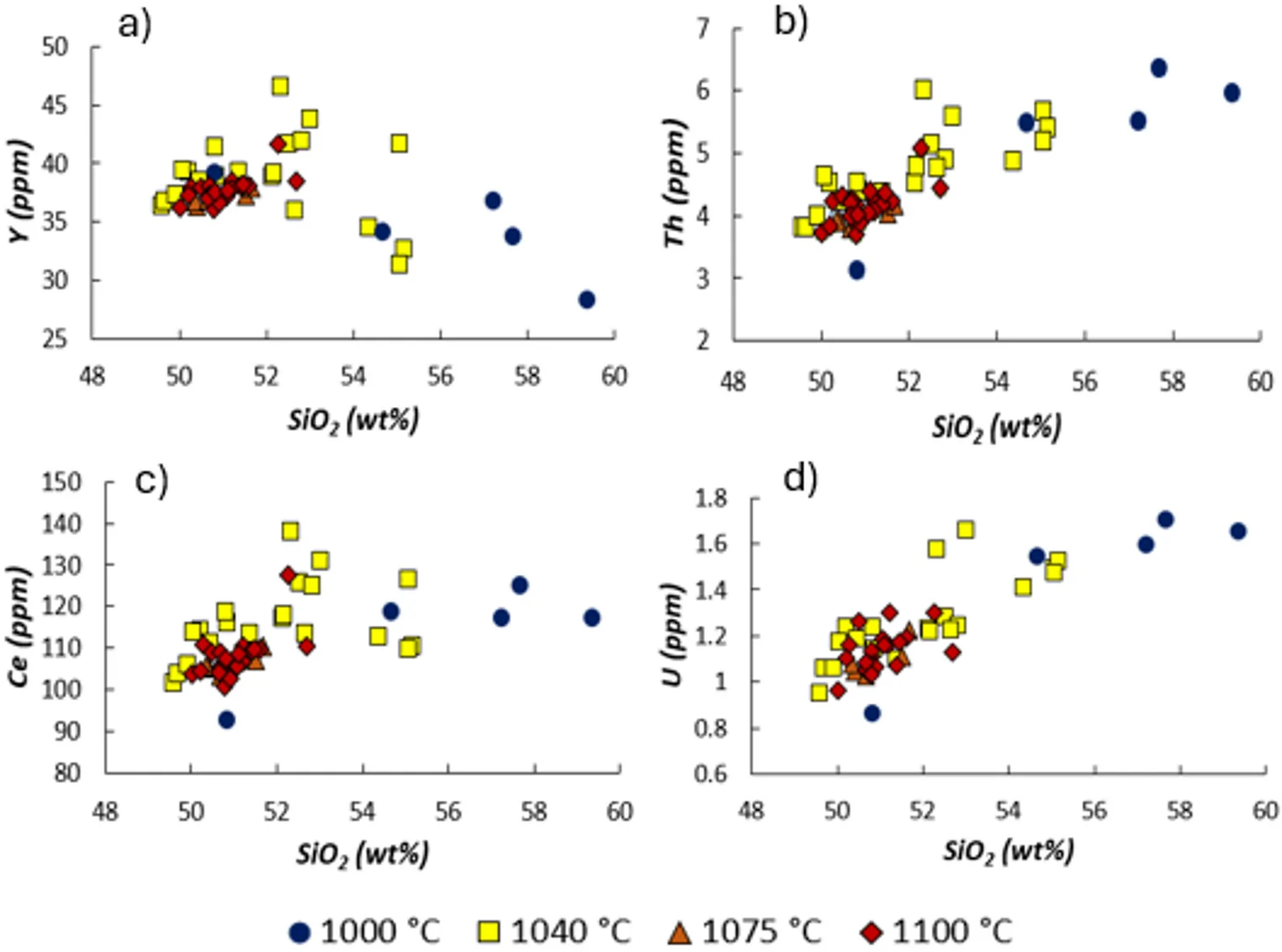
Evolution of trace elements contents with SiO_2_ of the residual glass. See text for explanation

Most analysed trace elements of residual glasses (Zr, U, Th, Ba, La) show broadly incompatible behaviour, as illustrated in Fig. 8 for Th and U, in accordance with the nature of crystallising phases which do not incorporate those elements. The contents of those incompatible elements increase by 30–50% between crystal-free and the most crystallised charges, in agreement with phase proportions (Table 3). The contents of Y and Ce show instead flat/decreasing trends with increasing SiO_2_, which reflects uptake of these elements by Amp (Y) or Ap (Ce) in low temperature (1000 °C) charges crystallising these minerals in abundance.

**Fig. 9:**
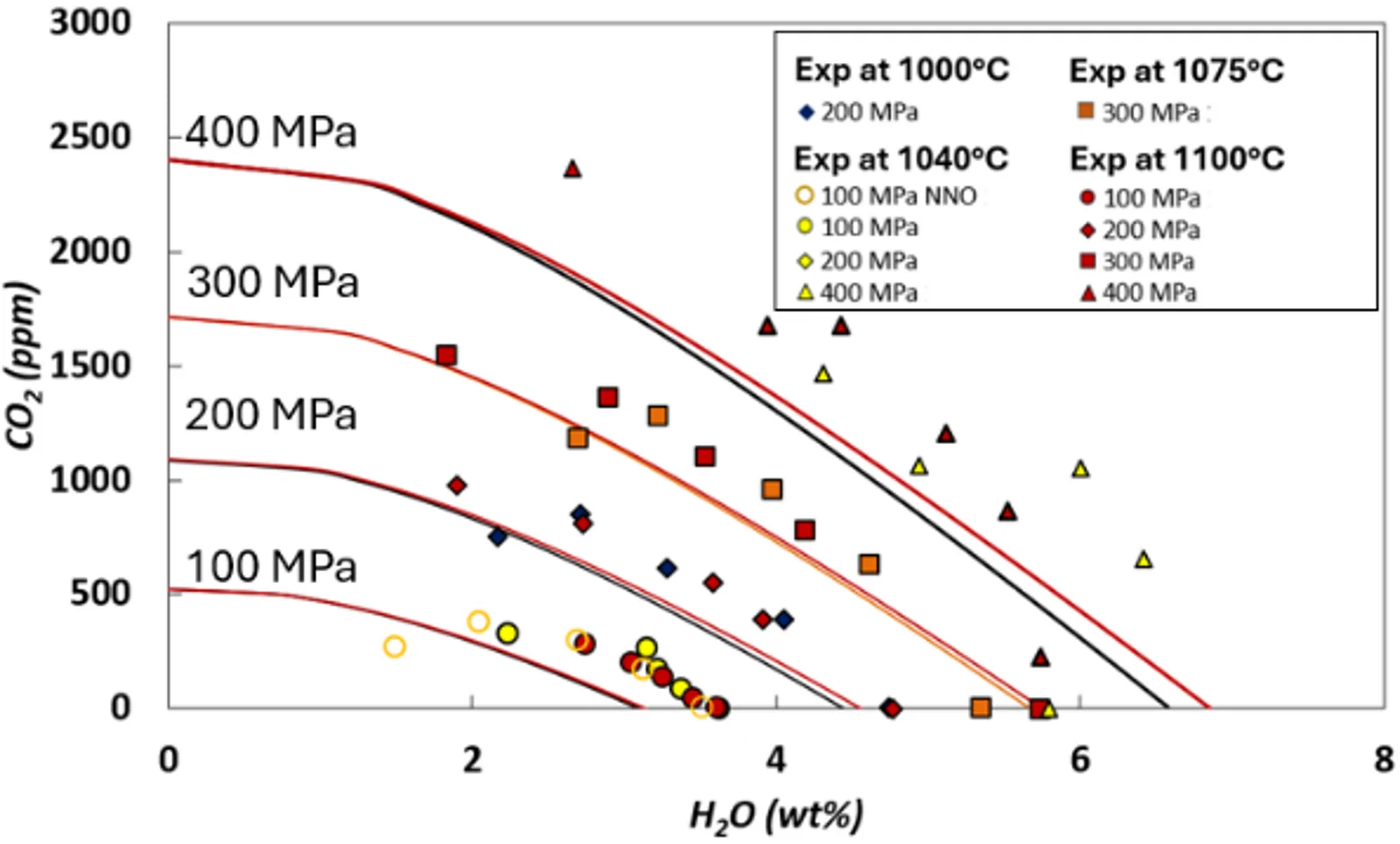
The H_2_O–CO_2_ plot of experimental charges compared with calculations made using the Volatilecalc model (Newman and Lowenstern 2002). Note that the experiments lie above the calculated trend. Model lines are red for models at 1100 °C, orange at 1075 °C, and black at 1040 °C

The experimental glasses display a systematic inverse relationship between H_2_O and CO_2_ contents, showing that the experimental protocol of loading different amounts of H_2_O and CO_2_ succeeded in controlling both species’ abundances in quenched glasses. The experimental data are plotted in Fig. 9, along with the calculated trends using the VolatileCalc model (Newman and Lowenstern 2002). It is apparent that the latter underestimates the CO_2_ content of experiments, emphasising the high sensitivity of CO_2_ to melt composition. The data also show that temperature has no appreciable effect on either H_2_O or CO_2_ solubilities in the temperature interval explored, since at any given pressure, the H_2_O–CO_2_ data gathered at different temperatures define a single trend within analytical error. See the supplementary information for comparisons with other generic solubility models (Behrens et al. 2009; Dixon et al. 1995; Ghiorso and Gualda 2015; Iacono-Marziano et al. 2012; Papale et al. 2006).

Given that the pressure estimates of the Nabro melt inclusions derive primarily from their CO_2_ content (Donovan et al. 2018), it is important to have a calibrated relationship between these two parameters. We have fitted our data on CO_2_ to the following empirical expression:

7 $$\begin{aligned} f{\mathrm{CO}}_{{\mathrm{2}}} \left( {{\mathrm{bar}}} \right) = &\,0.00{\text{161 }}( \pm 0.000{\mathrm{5}}) \times \left( {{\mathrm{CO}}_{{{\mathrm{2}} - {\mathrm{ppm}}}} } \right)^{{\mathrm{2}}}+ \\ & {\text{ 2}}.{\text{83 }}( \pm 0.{\mathrm{56}}){\text{ }} \times \left( {{\mathrm{CO}}_{{{\mathrm{2}} - {\mathrm{ppm}}}} } \right){\text{ }}\left( {{\mathrm{R}}^{2} = {\text{ }}0.{\mathrm{92}}} \right) \\ \end{aligned} $$

To extract *f*CO_2_ in our experiments, we assume that XH_2_O_fin_+XCO_2_ = 1, a reasonable approximation given the very low contents in Cl, F and S of the charges. The *f*CO_2_ of each charge is given by the standard relationship:

8 $$f{\mathrm{C}}{{\mathrm{O}}_{\mathrm{2}}}\,=\,{\mathrm{XC}}{{\mathrm{O}}_{\mathrm{2}}} \times ~{\gamma\mathrm{C}}{{\mathrm{O}}_{\mathrm{2}}} \times {\text{ }}{{\mathrm{P}}_{{\mathrm{tot}}}}$$

where γCO_2_ is the fugacity coefficient of pure CO_2_ at P and T, which is calculated using a Modified Redlich-Kwong (MRK) equation of state (Holloway 1977). The high correlation coefficient obtained shows that Eq. (7) correctly reproduces the experimental data and can be used to re-evaluate entrapment pressures of MI analysed in Nabro’s 2011 eruptive products (see below).

## Discussion

Early studies (Civetta et al. 1974; De Fino et al. 1978) suggested that some of the variations in the marginal zones of Afar might reflect mantle redox conditions, and indicated a substantial role for clinopyroxene in the crystallisation of magmas. More recent work (e.g. Rooney et al 2012; Thompson et al. 2015) has suggested that the mantle beneath Afar is anomalously hot and wet compared with other intraplate or divergent settings. Clinopyroxene is the liquidus phase under plausible conditions at Nabro (Donovan et al. 2018) and the primitive magma may contain around 1.3 wt% H_2_O dissolved in the melt (H_2_O_melt_), based on melt inclusion data. Redox conditions are inferred to lie in the vicinity of the Quartz-Fayalite-Magnetite (QFM) and Ni-NiO (NNO) buffers.

The petrological analysis (Donovan et al. 2018) identifies two batches of magma that were stored in the shallow crust at 5–7 km depth (150 ± 50 MPa)—one for an extended period and the second injected immediately before the eruption (based on textural differences) (Donovan et al. 2018). The glassy, fresh magma was referred to as “batch 1” and the older stored magma “batch 2”. The precise storage conditions in the shallow crust could not be established firmly on the basis of thermobarometry, however. As reviewed recently by Wieser et al. (2023), the application of different thermodynamic packages and geothermobarometers (in the present case, Cpx–liq, Pl–liq, apatite, Ol–melt) and Ilm–Mt pairs maps to a wide range of temperatures and pressure in most instances (at Nabro, 980–1120 °C and 200–500 MPa) (Fig. 2). Melt inclusions have H_2_O_melt_ = 0.5 to 2.1 wt% (average of 1.5 wt%) and CO_2_ contents of up to 3500 ppm. These volatile contents represent entrapment pressures from 20 to 500 MPa (using Newman and Lowenstern (2002), or Gualda and Ghiorso (2015) models) with two broad clusters at 150 ± 50 MPa and 300 MPa for batch 2 and 1, respectively. The average temperature from melt inclusions is 1080 °C with *f*O_2_ between QFM and NNO. In this section, we combine these results with our own.

### Experimental constraints on final storage conditions of 2011 magma at Nabro

Looking first at the 200 MPa phase diagram (Fig. 3), the pre-eruptive domain is essentially constrained by the occurrence of Pl, which is the third phase to crystallise in all our experiments, after Cpx and Ol, and is an abundant phase in the natural samples. Near liquidus co-precipitation of these phases, as required by the crystal-poor character of the batch 1 magma, is possible over a wide and inversely correlated range of T–H_2_O_melt_ values: from 1100 °C at 1 wt% H_2_O_melt_ down to 1010 °C at 4.5 wt% H_2_O_melt_. Consideration of the rim compositions of both Ol (Fo_70_) and Pl (An_60_) phenocrysts significantly restricts the range of values, essentially to 1025 ± 10 °C and 3.5–4 wt% H_2_O_melt_ (Fig. 3). Simultaneous solution of Eqs. (3) and (5) using the average rim compositions Fo_70_ and An_60_ gives T = 1021 °C and H_2_O_melt_ = ~ 4 wt%. Use of Eq. (6) with 4 wt% H_2_O_melt_ gives a temperature of 1018 °C for the MgO-rich matrix glass composition reported by Donovan et al. (2018), and 992 °C for the MgO-poor one.

Altogether, these temperatures are consistent with the pre-eruptive temperature inferred by Donovan et (2018), which was based on an ensemble of MI analyses and used a variety of thermometers (Fig. 2), but at the lower end of the range. Given that Fo content depends on *f*O_2_, this could reflect in part that the Nabro magma was slightly more oxidised (i.e. at QFM + 0.4). Using the same petrological arguments (An, Fo and crystal contents), the polybaric section at 1040 °C constrains the final pressure of magma storage to be around 100–200 MPa (Fig. 4a). For a crustal density of 2780 kg m^− 3^ (Makris and Ginzburg 1987), this storage zone lies at around 7–10 km, consistent with the findings of Hamlyn et al. (2014) and Gauntlett et al. (2023).

Donovan et al. (2018) described some reverse-zoned Ol and Pl crystals (from Fo_70_–_75_ and An_60–65_ to An_70–75_), which suggests that some magma parcels may have experienced transient storage at ∼ 100 MPa and 1075–1040 °C (Fig. 3). An increase in *f*O_2_ in the reservoir or during magma ascent could also explain the inverse trend recorded by Ol, but not that of Pl. The data on Mt (see SI) suggest instead that the *f*O_2_ of the magma decreased during the last stage of evolution. Of note, Pl with An > 80, and Ol with Fo > 80 have not been produced in our experiments, suggesting they come from a more mafic magma, as addressed in the next section.

Therefore, consistent with the work of Donovan et al. (2018), the experiments suggest that the primary magma storage region for this eruption was < 250 MPa. Most of our experiments were wetter than the most water-rich inclusions (2.1 wt%), and they collectively indicate that the last equilibration occurred in a H_2_O-richer environment than the one recorded by the majority of MI (see next section for further discussion). The fact that some older rocks contain Amp suggests that conditions favourable to Amp crystallisation (i.e. H_2_O-rich) can indeed be reached in the plumbing system of Nabro (essentially T < 1030 °C, Fig. 3).

### Additional constraints from melt inclusions

In all panels of Fig. 7, the curved trend defined by the MI population is perpendicular to any of those defined by experiments run at constant T but variable H_2_O_melt_. Overall, for most oxides, the bottom part of the MI trend joins the dry end of the trend defined by charges at 1040 °C (as indicated by arrows for TiO_2_, CaO and MgO). This suggests that the MI trend reflects in part decreasing temperature at somewhat increasing H_2_O_melt_. For all oxides, the 1000 °C trend lies significantly below the lower end of the MI trend, showing that this temperature has not been reached by magmas erupted in 2011. Some of the higher-pressure higher-temperature experiments also overlap with higher MgO melt inclusion compositions, but the 400 MPa–1040 °C experiments produce melts too poor in MgO.

Our dataset allows us to recalculate the entrapment pressures of MI analysed by Donovan et al. (2018). An advantage of our procedure is that both the experimental and natural glasses have been analysed using the same instrument and standards, which considerably limits error propagation issues when using general solubility models, as illustrated above and further discussed below. To do so we use the empirical Eq. (7) to extract *f*CO_2_ from dissolved CO_2_. For H_2_O we use the solubility model of Jiménez-Mejías et al. (2021), calibrated on alkali-rich basalts, to calculate the corresponding *f*H_2_O:

9 $$f{{\mathrm{H}}_{\mathrm{2}}}{\mathrm{O}}\,=\,{\mathrm{11}}.{\text{5813 }} \times {\text{ }}{{\mathrm{H}}_{\mathrm{2}}}{{\mathrm{O}}_{{\mathrm{melt}}}}{\left( {{\mathrm{wt}}\% } \right)^{{\mathrm{1}}.{\mathrm{84}}0{\mathrm{7}}}}$$

Note that unlike CO_2_, H_2_O solubility in basaltic melts is little affected by changes in melt composition, and the use of other H_2_O solubility models would be inconsequential as far as pressure of entrapment is concerned. The calculated *f*H_2_O corresponding to Nabro’s MI suite falls mostly in the range 50–300 bar, while *f*CO_2_ goes up to over 20 kbar for the most CO_2_–rich inclusions. This shows clearly that what matters in calculating the pressure of MI entrapment at Nabro is their amount of CO_2_. The pressures of entrapment are calculated assuming that:

10 $${{\mathrm{P}}_{{\mathrm{tot}}}}={\text{ }}{{\mathrm{P}}_{{\mathrm{H2O}}}}+{{\mathrm{P}}_{{\mathrm{CO2}}}}$$

where P_i_=X_i_ × P_tot_, i standing for either H_2_O or CO_2_. For each couple of H_2_O–CO_2_ data, the calculation is performed iteratively until the condition X_H2O_+X_CO2_=1 is fulfilled. Saturation pressures and temperatures were computed with a Monte Carlo scheme that propagates analytical and calibration uncertainties simultaneously. For each melt inclusion, 1000 realisations were drawn. Major-element oxides were perturbed using Gaussian distributions with 1σ analytical uncertainties (0.01–0.1 wt%, oxide-dependent), and H₂O and CO₂ were perturbed using the sample-specific uncertainties reported for each measurement. All compositional draws were truncated at zero by rejection rather than clipping, so that the truncated distributions retain unbiased moments; for samples in which the H₂O content lies at or below its own analytical uncertainty, the value was held fixed instead, since truncation would otherwise displace the mean upward.

Thermometer coefficients were drawn jointly from a multivariate normal distribution defined by the calibration covariance matrix. This step is essential: the regression coefficients are strongly correlated (*r* = − 0.91 between the intercept and the Mg# term), and drawing them independently inflates the propagated temperature uncertainty by more than an order of magnitude. An additional independent term representing the residual scatter of the calibration (1σ = 14.7 °C) was added to each temperature realisation, so that the reported uncertainty reflects the error of prediction for a new sample rather than only the uncertainty in the position of the regression surface. The two coefficients of the CO₂ fugacity expression were drawn independently.

Because the thermometer depends on pressure and the saturation pressure depends on temperature, each realisation was solved by outer iteration between the two until pressure converged to within 1 bar. Total pressure was obtained at fixed temperature by bisection on the condition X_H₂O_ + X_CO₂_ = 1, with fugacity coefficients from an MRK equation of state (Holloway 1977).

**Fig. 10:**
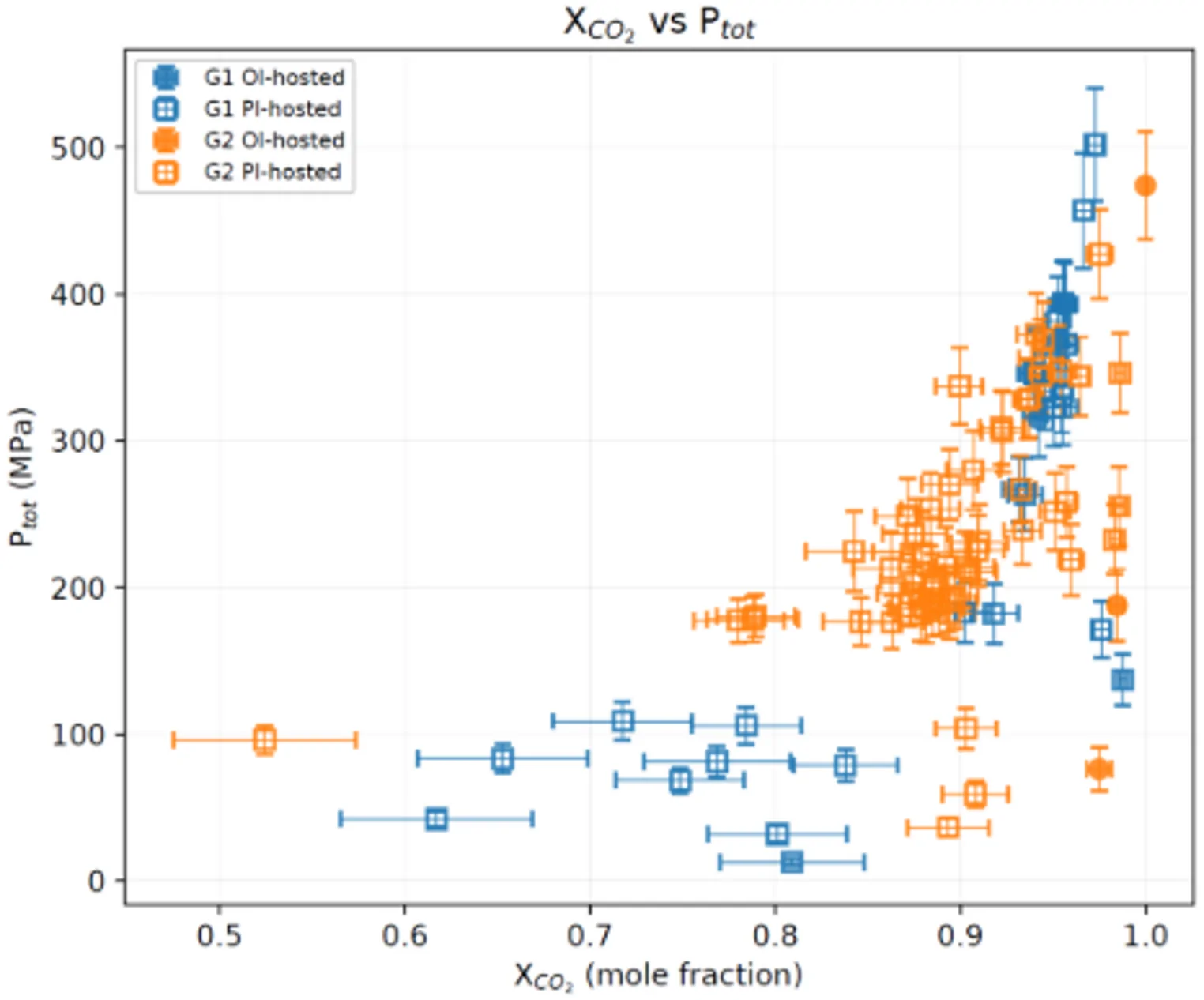
Evolution of the mole fraction of CO_2_ of the excess fluid with pressure, calculated for the MI analysed by Donovan et al. (2018), using the empirical CO_2_ solubility model developed in this work (Eq. 7). G1 and G2 refer to the groups identified in Donovan et al. (2018). Closed symbols are olivine-hosted inclusions, and open symbols are inclusions in plagioclase

The results are shown in Fig. 10, in which we distinguish MI from batch 1 from those of batch 2 in a X_CO2_ vs. P_tot_ diagram. We also distinguish MI trapped in Ol from those in Pl. MI in Batch 1 define a nearly vertical trend, which starts at around 500 MPa, with fluids having X_CO2_>0.95. At lower pressures, the fluid gets more H_2_O-rich, notably around 100 MPa. Batch 2 shows broadly the same pattern, except that the spread in X_CO2_ is wider at higher pressure.

Although data are few, MI in Ol define a near vertical trend with little spread in X_CO2_, as expected from degassing upon decompression in a closed system. In contrast, MI in Pl, though starting at equally high pressures as those in Ol, display a larger spread in X_CO2_, widening as pressure decreases. This suggests either magma stalling and crystallisation at various levels, enriching the residual melt in H_2_O, or decompression with different amounts of excess fluid: the more excess fluid the magma has at high pressure, the less steep is the decompression path (e.g., Dixon and Stolper 1995). Regardless, that MIs in Pl record pressures as high as those in Ol indicates that the magma column beneath Nabro is saturated with these two minerals over a significant thickness of the crust. As Batch 2 displays a pressure spread similar to that of Batch 1, it suggests also that the resident magma has captured Pl from Batch 1, or from previous recharge events.

**Fig. 11:**
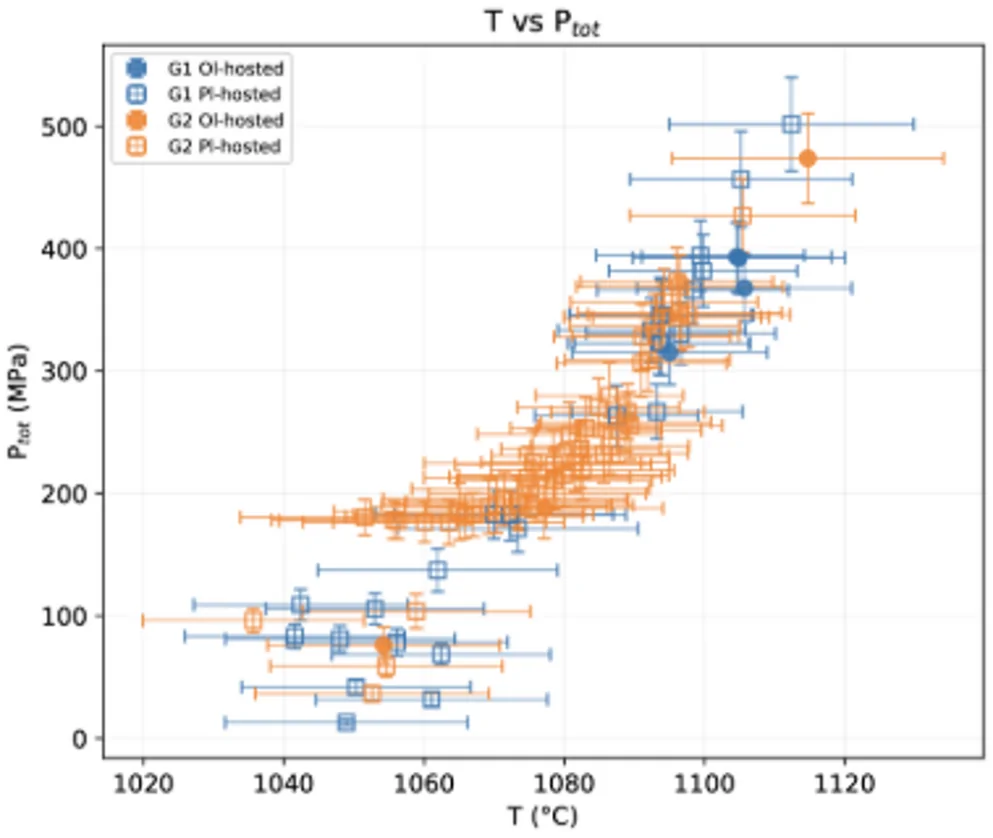
Correlation between the pressure of MI entrapment, as calculated from CO_2_-H_2_O fluid saturation, and melt temperature as calculated from Eq. (6). G1 and G2 refer to the groups identified in Donovan et al. (2018). Closed symbols are olivine-hosted inclusions, and open symbols are inclusions in plagioclase. See text for discussion

An additional calculation can be done with our experimental data, by using Eq. (6) which relates melt composition to temperature, pressure and H_2_O_melt_. Assuming that all MI trapped in Ol and Pl phenocrysts grew from a magma composition not far from that used in our experiments, we calculate the temperatures of MI entrapment, using the pressures as calculated above. Temperatures range from 1010 °C up to 1130 °C, displaying a strong positive correlation with pressure (Fig. 11). The trend is almost continuous, though there are clusters in the intervals 200–300 MPa and 300–400 MPa, and around 100 MPa. There is no obvious difference, however, between batch 1 and batch 2 MIs. Interestingly, the lower temperature end of the trend falls close to the pre-eruptive P-T field inferred from phase equilibrium arguments above. The general continuity of data shows that decompression of magma at Nabro accompanies cooling. This in turn suggests that MI are entrapped during magma stagnation and crystallisation at various levels in the mid to upper crust.

**Fig. 12:**
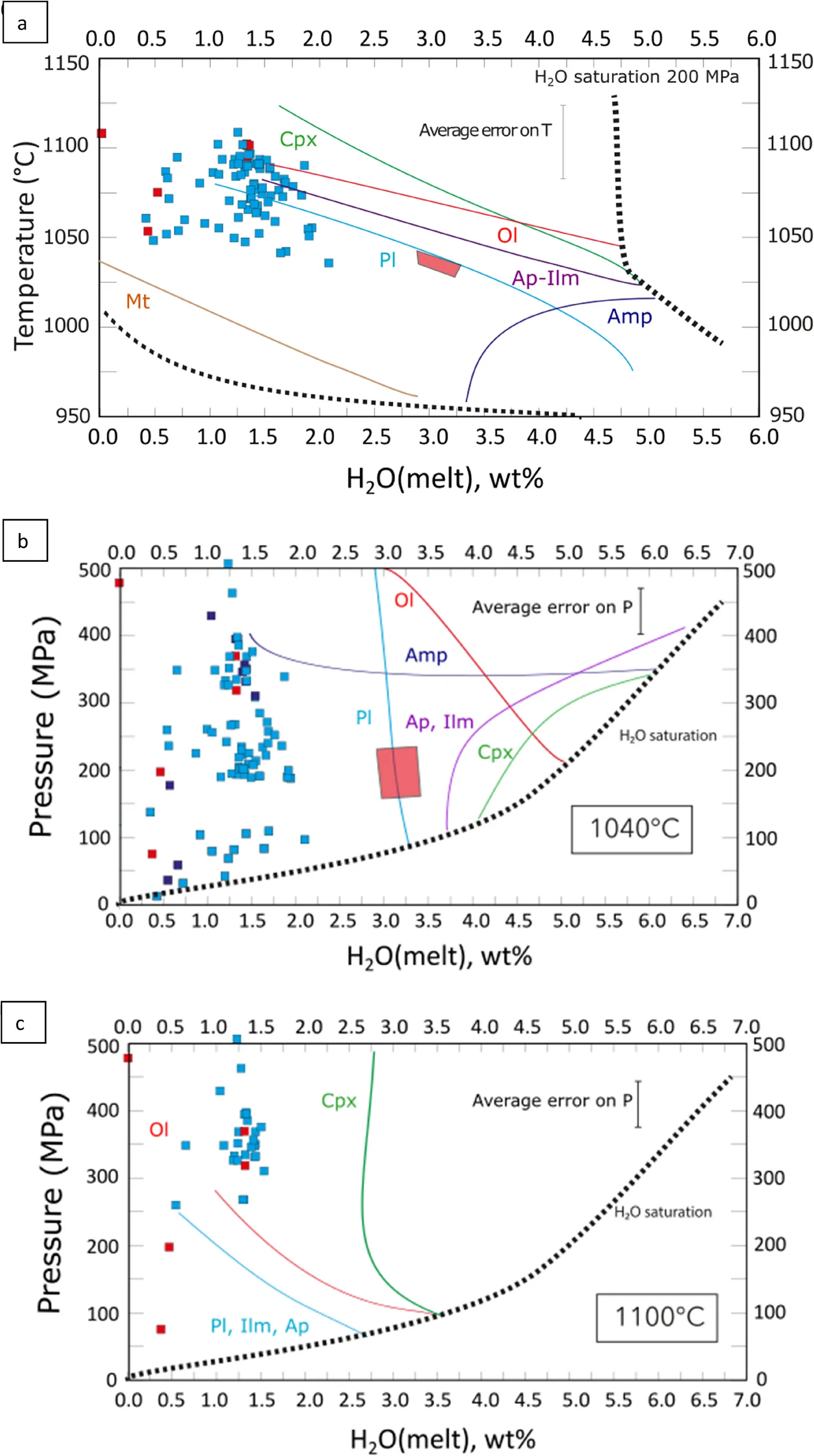
Correlation between the H_2_O_melt_ of MI, as measured by SIMS, and their temperature (panel **a**) and pressure (panels **b**, **c**) of entrapment, superimposed of the phase relationships of Figs. 3 and 4. Red squares for MI in Ol, blue squares for MI in Pl. In (**b**) all MI have T < 1080 °C, and dark blue squares are for MI with T near 1040 °C. In (**c**) all MI have T > 1080 °C

Further insight into the status of MI in phenocrysts is provided by plotting the temperature-temperature pairs calculated for each MI in the experimental phase diagrams. When plotted in the 200 MPa section (Fig. 12a), taking into account the error on T, it appears that the majority of MI in Pl plots within the Pl stability field or close to it. Similarly, three out of five MI hosted by Ol do not fall within the Ol stability field, yet are close to it, if proper consideration is given to error on T. Overall, the MI population is situated somewhat away from pre-eruptive conditions. When plotted in the polybaric-1040 °C section, although falling within the stability fields of either Pl or Ol (Fig. 12b), the MI define an array that does not trend toward the pre-eruptive domain.

Filtering out all MI in Pl with temperatures lower than 1080 °C and plotting those remaining in the polybaric section at 1100 °C (Fig. 12c), shows that almost all MI in Pl fall largely outside the Pl stability field at this temperature. This last observation shows that a significant proportion of MI and of their crystal hosts did not grow from their host magma but are more likely sourced from a more mafic body, parental to the bulk compositions erupted in 2011. Considering the spread of “hotter” MI in Pl suggests that such a more mafic parent may have predominantly stalled and crystallised in the pressure interval 300–400 MPa (Fig. 12c).

### Temperature change during decompression

The phase relations superimposed on the polybaric diagrams are drawn at constant temperature, which requires an estimate of the temperature change a magma experiences on ascent. For a purely adiabatic (isentropic) decompression of the silicate liquid, the temperature gradient is given by (∂T/∂P)_S = αT/(ρC_P). Using values appropriate for a basaltic liquid at ~ 1050 °C (α ≈ 5 × 10⁻⁵ K⁻¹, ρ ≈ 2700 kg m⁻³, C_P ≈ 1500 J kg⁻¹ K⁻¹), this yields roughly 1.6 °C per 100 MPa, so ascent from 500 to 100–200 MPa produces only about 5–15 °C of cooling. This is smaller than the calibration uncertainty of the thermometer (± 16–20 °C), and the constant-temperature assumption is therefore justified for the adiabatic component alone.

The adiabatic term is, however, unlikely to dominate the real thermal budget. The decreasing CO₂ mole fractions of the inclusions (X_CO₂_ = 0.94–1.00) with pressure (Fig. 10) suggests extensive volatile exsolution during ascent. The expansion of the exsolving fluid drives additional cooling of several tens of degrees, depending on the exsolved fluid mass and on whether degassing is open or closed. This is opposed by the latent heat released during crystallisation (~ 400 kJ kg⁻¹), which for a basalt can offset several tens of degrees for only a few percent crystallisation; degassing-induced dehydration raises the liquidus and promotes precisely this crystallisation, so the two effects are coupled. The net temperature change therefore reflects a competition between fluid expansion and latent-heat release and cannot be resolved without modelling the degassing path explicitly (e.g. with an isentropic Rhyolite-MELTS calculation); this would require a knowledge of the bulk fluid content at the onset, which is difficult to constrain.

Because the retrieved temperatures and pressures are available for every inclusion, the slope of the observed T–P array (about 60 °C decrease in the pressure interval 100–500 MPa, Fig. 11) provides an empirical, assumption-free check on the ascent path, although it is likely blurred by the inclusions deriving from more than one magmatic episode. Considering Nabro’s context (Fig. 11), we suggest that Fig. 12c is appropriate for decompression steps occurring at high pressure, while Fig. 12b better captures the low-pressure part of the decompression path.

Clearly, the inferred storage region does not explain the full range of mineral compositions encountered at Nabro. What it does suggest is that the dominant mineral assemblage is stable below ~ 200–250 MPa, and around 1020–1030 °C, with rather elevated H_2_O_melt_ for this magmatic and tectonic setting (see also Sauvalle et al. 2024). The water content preserved by the MI is < 2 wt% (Donovan et al. 2018), which is lower by 1–2 wt% relative to that indicated by phase equilibria. This either reflects provenance of MI from more mafic and drier magmas, as suggested above, or post-entrapment loss of H_2_O from MI (of about 1 wt% H_2_O_melt_ for the H_2_O-rich MI, which would decrease calculated temperatures by about 10 °C). Whatever the reason, the main lesson is that a significant, if not dominant, proportion of MI at Nabro does not inform the shallow P–T conditions immediately preceding eruption (as reproduced experimentally).

**Fig. 13:**
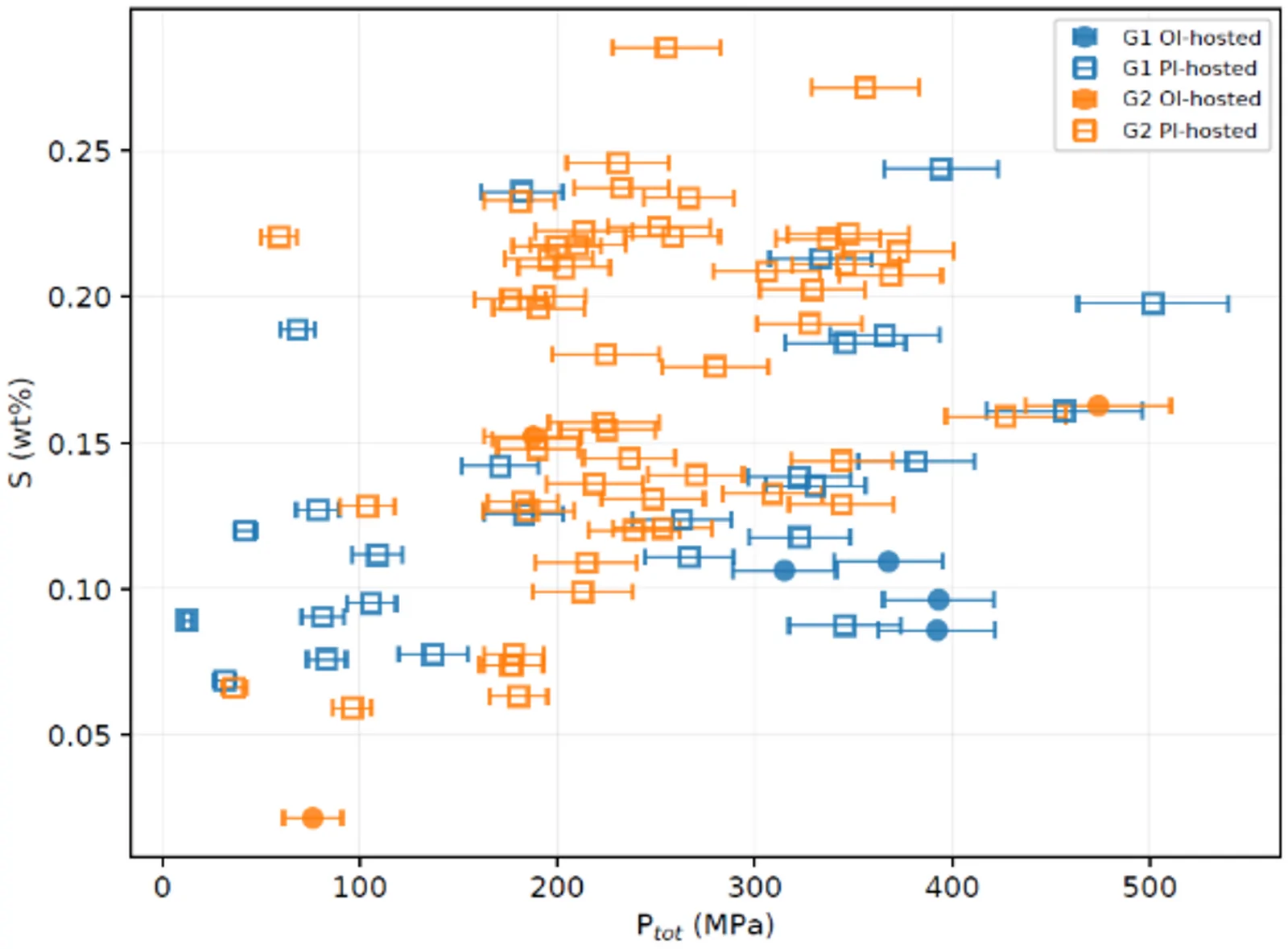
Relationships between the S content of MI and their pressure of entrapment. G1 and G2 refer to the groups identified in Donovan et al. (2018). Closed symbols are olivine-hosted inclusions, and open symbols are inclusions in plagioclase

While our starting material is from batch 2, the bulk compositions of both batches of magma that were discussed by Donovan et al. (2018) are very similar. The key differences between the batches are in their H_2_O and S contents, the Mg# of the inclusions, and the magma crystallinity. Batch 2 is more crystalline, had higher H_2_O and S, and lower Mg# consistent with storage in the crust. There is, however, evidence for crystal exchange between the batches, as inferred above. The An content of Pl is generally correlated with S and negatively correlated with H_2_O. The S contents of MI define a very broad positive correlation with P_tot_, again with no obvious difference between the two batches (Fig. 13).

Our experimental data are consistent with the interpretation that batch 2 may have stalled in the shallow crust and increased its H_2_O_melt_ due to isobaric crystallisation, probably at somewhat lower temperatures than previous estimates (Donovan et al. 2018). The highest Mg# inclusions were in batch 1, and our data suggest that these may have been trapped at high pressures (> 400 MPa).

Older lavas sampled at Nabro also show considerable heterogeneity and the presence of xenocrystic material. The zonation of the crystals is consistent with an eruption that occurred following the second of two intrusive episodes, but the data gathered here and by Donovan et al. (2018) collectively suggest that there are multiple cooling reservoirs beneath the volcano that at least some of the intrusions encountered as they rose to the surface.

### Implications for magma degassing and sulphur budget

**Fig. 14:**
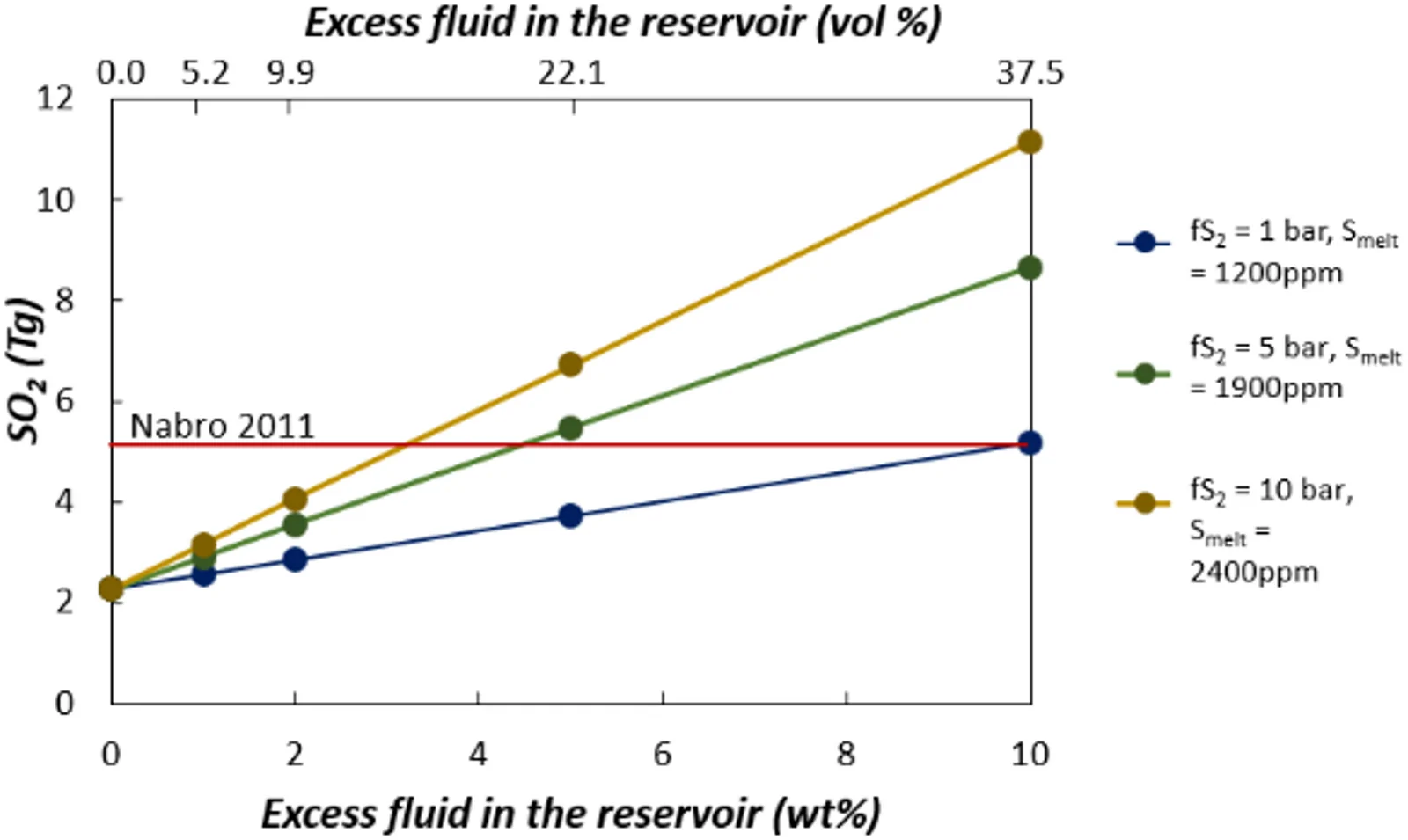
Relationships between the excess fluid in the shallow reservoir at Nabro (100–200 MPa) and the SO_2_ yield calculated for a residual S content in the matrix glass of 500 ppm, and three values of *f*S_2_. The horizontal red line corresponds to the SO_2_ yield as measured by satellite (Theys et al. 2013). The upper X axis shows the correspondence in terms of volume of exsolved fluid, calculated using a mean density for fluid of 0.5 gr/cm^3^ and of 2.6 gr/cm^3^ for crystals+melt

The Nabro eruption ejected an estimated 4.5 Tg of SO_2_ (Theys et al. 2013). The volume of emitted magma is estimated to be around 0.5 km^3^ (DRE). The average S content of MI is 1550 ± 570 ppm. Taking this value and the residual S content of matrix glass (about 500 ppm, Donovan et al. 2018), indicates degassing of 1.1 Tg S, or 2.2 Tg SO_2_, hinting at a deficit of S, though less severe than has been shown for more silicic eruptions (e.g., Westrich and Gerlach 1992). In the following we evaluate whether an excess fluid phase could explain such a S deficit. We use the pre-eruptive conditions as estimated above, and the S solubility model of Lesne et al. (2015) calibrated for alkali-rich basaltic magmas, which relates the S content of the melt to the fugacities of both H_2_S and SO_2_. We perform the calculations at 200 MPa and a temperature of 1030 °C, using an H_2_O_melt_ of 3 wt%. The simulations are made at QFM–NNO, with the same procedure used for H_2_O and CO_2,_ as explained above, expanding Eq. (10) to include P_H2S_ and P_SO2_. An unknown is the value of *f*S_2_, which is varied in our calculations so as to give S contents in the range 1000–2400 ppm, in accord with the MI record (Fig. 12). An additional unknown is the amount of excess fluid in the reservoir, which is a variable parameter in our calculations. The results are shown in Fig. 14.

Results are shown for three values of *f*S_2_ and excess fluids up to 10 wt%. Note that we do not assume a fixed P_SO2_: total SO_2_ yield is assumed to include S from SO_2_, H_2_S and S in the melt, all of which are converted to SO_2_ on full degassing. To reproduce the SO_2_ yield measured by satellite (Theys et al. 2013), a magma with an *f*S_2_ of 1 bar would need an excess fluid of 8 wt%, and the average melt S content would be slightly over 1000 ppm. For an *f*S_2_=5 bar, the amount of excess fluid needed to match the satellite yield drops to about 3 wt%, and the melt in the shallow reservoir would have 1900 ppm dissolved S. Increasing *f*S_2_ to 10 bar would further diminish this excess fluid to slightly above 2 wt% with a S melt content of 2400 ppm. All three scenarios are compatible with the range of measured S contents in MI (Fig. 13).

Taking the intermediate case as representative of average pre-eruptive conditions suggests that about 3 wt% of an excess fluid was present in the shallow reservoir, holding about half of the total budget of S released to the atmosphere. If the pre-eruptive melt S content was instead around 1000 ppm, this would more than double the amount of excess fluid (8 wt%). Such a large fraction, although common for silicic magmas, seems also to hold for basaltic magmas. A similar value has been indeed inferred, for instance, for the 2021 eruption at La Palma (Burton et al. 2023; Frascerra et al. 2024), and elevated fluid contents have been proposed for other basaltic volcanoes (Stromboli, Etna; (Allard 2010).

In the case of Nabro, this excess fluid is of relevance for understanding the plume dynamics of the 2011 event. In particular, it may well have contributed to the explosive character of the Nabro event, which was able to propel S into the stratosphere (Clarisse et al. 2014; Fromm et al. 2014; Vernier et al. 2013), perhaps assisted by convective transport linked to the Asian monsoon (Bourassa et al. 2012; 2013). Indeed, the upper scenarios considered here (~ 8 wt% excess fluid) would mean that the magma had ~ 30 vol % fluid prior to eruption (Fig. 14).

## Conclusions

We present experimental phase equilibrium results for the 2011 Nabro products. These suggest that the magma was stored in the shallow crust at pressure < ~ 200–250 MPa and temperature around 1020–1040 °C prior to the eruption, most likely at *f*O_2_ ~QFM. This is equivalent to the ~ 7 km depth magma chamber modelled by Hamlyn et al. (2014).

Prior to the eruption, there were likely at least two intrusive events that brought magma to shallow depths, the first around the time of a local earthquake in March 2011, and the second just before the eruption. These are associated with the compositions of the smaller phenocrysts and the rims of the older phenocrysts, which are reproduced experimentally here.

The primitive phenocryst cores were not reproduced in the experiments, but plagioclase was produced at high pressures in drier experiments, which may suggest that these crystals formed deeper in the system, perhaps from a more mafic melt. This would be consistent with the melt inclusion record (Donovan et al. 2018). We combined the experimental results with the melt inclusion data to recalculate the entrapment pressures of the melt inclusions analysed by Donovan et al. (2018), and developed a solubility model for the Nabro 2011 magmas. We observe that while many of the melt inclusions are consistent with a storage region at around 1040 °C, they are typically drier than the final storage region as inferred from the experiments—and higher temperature inclusions are not consistent with their carrier magma, also suggesting that they may have come from a hotter, drier melt at depth. The MI reflect a longer history of the magma as it rose through the complex plumbing system at Nabro.

We further use the data to calculate that there was likely ~ 3 wt% excess fluid in the magma chamber at the time of eruption, which may explain its explosivity and the large associated sulphur release. The presence of large amounts of excess fluid at basaltic volcanoes, perhaps particularly those that are long quiescent, has implications for hazard assessment as it may enhance the explosivity of eruption.

## Supplementary Information

Below is the link to the electronic supplementary material.

Supplementary Material 1

Supplementary Material 2

## Data Availability

The data underlying this article are available in the article and in its online supplementary material.
