# Supplementary material for: Experimental constraints on pre-eruptive conditions at Nabro volcano, Eritrea, 2011, and implications for sulphur emissions
Source: Contrib Mineral Petrol. 2026 Sep 4;181(10):76. doi: 10.1007/s00410-026-02357-2 (PMC13545127; doi:10.1007/s00410-026-02357-2)
Supplement: Supplementary file 1 [file 410_2026_2357_MOESM1_ESM.docx]

# Supplementary data: Figures and sample descriptions

This document contains summary tables and figures; full experimental data and standards are in the supplementary spreadsheet.

Contents

[Supplementary data: Figures and sample descriptions	1](#_Toc227941474)

[Mineralogy and textures	1](#_Toc227941475)

[Olivine	1](#_Toc227941476)

[Clinopyroxene	2](#_Toc227941477)

[Fe-Ti oxides	3](#_Toc227941478)

[Apatite	4](#_Toc227941479)

[Plagioclase	4](#_Toc227941480)

[Additional H_2_O-CO_2_ models	6](#_Toc227941481)

[Assessing the temperature model (equation 6)	8](#_Toc227941482)

## Mineralogy and textures

The petrography of the products was described in Donovan et al. (2018), so here below we summarize the main petrological findings of this study and provide new compositional data concerning main phenocryst phases in the accompanying spreadsheet.

According to Donovan et al. (2018) two different batches of magmas were involved in the eruption. The mineral assemblage consists of plagioclase, olivine, clinopyroxene, magnetite, ilmenite and minor fluorapatite in both batches – although batch 1 tends to be more plagioclase-rich relative to the other phases. The batches mostly differ on the crystal content; batch 1 being crystal poor (< 15%) and batch 2 having up to 70% of crystals.

Here, we present mineral data from the whole suite of samples from the 2011 eruption, including lavas, tephras and bombs.

### Olivine

According to punctual analysis (Donovan et al. (2018)) and compositional profiles performed in this study, olivine phenocrysts are mostly normally zoned from cores at Fo 75–88 (majority 80-88) to rims Fo 70–75, though some are unzoned (Fo 64–85) and a few are slightly reverse-zoned (Fo ~57 to Fo 70). There is a wide range of compositions, particularly in samples obtained from lava flows, where some phenocrysts have much lower Fo contents (as low as Fo 50) than the phenocrysts obtained from tephra samples. The differences are also present in the microlites, with microlite cores from the tephras being higher in Fo (68–77, as opposed to 50–77 in the lavas). Hence, Fo <68 microlites thus seem to represent crystallization conditions in the upper conduit or during lava flow emplacement during the eruption. Thus, these compositions are not further considered.

The highest Fo80–88 phenocrysts cores we take as forming in the deeper system and likely entrained in the magma –this being evidenced by the presence of lower Fo70 rims on many though not all of them. The unzoned Fo–88 crystals are evidence that at least some of the magma erupted very quickly after rising from depth. Thus, the final equilibration episode is represented in the tephra by the presence of unzoned crystals and outer olivine rims at Fo 68–74 (see figure A1). This is the composition that is presented in Table 3 as representing the magma for the experiments.

Figure A1 shows several olivine crystal transects measured by electron microprobe. This demonstrates that while there are different starting compositions, the process that led to the zoning is similar in the different crystals.

| Distance from rim (µm) | Fo content |
| --- | --- |
| 230 | 81.7786 |
| 224.1 | 81.3404 |
| 218.2 | 81.0381 |
| 212.3 | 81.0995 |
| 206.4 | 81.0228 |
| 200.5 | 80.82 |
| 194.6 | 80.905 |
| 188.7 | 81.1777 |
| 182.8 | 80.9729 |
| 176.9 | 81.1388 |
| 171 | 80.8232 |
| 165.1 | 81.2237 |
| 159.2 | 81.1048 |
| 153.3 | 81.3644 |
| 147.4 | 81.2113 |
| 141.5 | 81.2395 |
| 135.6 | 81.3037 |
| 129.7 | 81.2763 |
| 123.8 | 81.4443 |
| 117.9 | 81.6911 |
| 112 | 81.3333 |
| 106.1 | 81.7462 |
| 100.2 | 81.6287 |
| 94.3 | 81.3432 |
| 88.4 | 81.8958 |
| 82.5 | 81.9689 |
| 76.6 | 82.1278 |
| 70.7 | 81.8411 |
| 64.8 | 82.2901 |
| 58.9 | 82.2102 |
| 53 | 81.9981 |
| 47.1 | 81.9686 |
| 41.2 | 81.3853 |
| 35.3 | 79.9563 |
| 29.4 | 79.2555 |
| 23.5 | 76.5307 |
| 17.6 | 74.2799 |
| 11.7 | 72.8934 |
| 5.8 | 72.7521 |
| 0 | 71.9781 |

| Distance from rim (µm) | Fo content |
| --- | --- |
| 94 | 82.234 |
| 89.1 | 82.1229 |
| 84.2 | 82.2978 |
| 79.3 | 82.4326 |
| 74.4 | 82.5236 |
| 69.5 | 82.1659 |
| 64.6 | 82.0463 |
| 59.7 | 82.2878 |
| 54.8 | 81.8907 |
| 49.9 | 80.7822 |
| 45 | 80.3859 |
| 40.1 | 80.0362 |
| 35.2 | 78.7362 |
| 30.3 | 76.9414 |
| 25.4 | 76.1403 |
| 20.5 | 74.9204 |
| 15.6 | 73.8642 |
| 10.7 | 73.0592 |
| 5.8 | 72.7996 |
| 0 | 72.4108 |

| Distance from rim (µm) | Fo content |
| --- | --- |
| 91.76 | 87.4615 |
| 86.95 | 87.4535 |
| 82.14 | 87.1942 |
| 77.33 | 87.1726 |
| 72.52 | 87.0897 |
| 67.71 | 86.8558 |
| 62.9 | 86.8189 |
| 58.09 | 86.2353 |
| 53.28 | 85.5121 |
| 48.47 | 84.7232 |
| 43.66 | 83.7728 |
| 38.85 | 82.8748 |
| 34.04 | 80.7694 |
| 29.23 | 79.393 |
| 24.42 | 77.7648 |
| 19.61 | 76.6396 |
| 14.8 | 75.1503 |
| 9.99 | 74.0056 |
| 5.18 | 72.4178 |
| 0.37 | 70.2961 |

| Distance from rim (µm) | Fo content |
| --- | --- |
| 92.09 | 87.2755 |
| 87.26 | 87.1343 |
| 82.43 | 86.8603 |
| 77.6 | 86.3436 |
| 72.77 | 86.4476 |
| 67.94 | 86.0397 |
| 63.11 | 85.5663 |
| 58.28 | 84.5067 |
| 53.45 | 83.6346 |
| 48.62 | 82.5774 |
| 43.79 | 81.5975 |
| 38.96 | 79.9429 |
| 34.13 | 79.227 |
| 29.3 | 78.1289 |
| 24.47 | 76.8998 |
| 19.64 | 75.1674 |
| 14.81 | 73.9189 |
| 9.98 | 72.9846 |
| 5.15 | 72.0043 |
| 0.32 | 68.1145 |

| Distance from rim (µm) | Fo content |
| --- | --- |
| 39 | 79.034 |
| 34.7 | 78.3865 |
| 30.4 | 78.0075 |
| 26.1 | 76.5916 |
| 21.8 | 75.2657 |
| 17.5 | 73.6064 |
| 13.2 | 72.8178 |
| 8.9 | 72.0581 |
| 4.6 | 71.699 |
| 0.3 | 66.0503 |

| Distance from rim (µm) | 61T1 |
| --- | --- |
| 180.5 | 79.846 |
| 171 | 80.0193 |
| 161.5 | 79.6726 |
| 152 | 79.6659 |
| 142.5 | 79.9849 |
| 133 | 80.2782 |
| 123.5 | 80.1354 |
| 114 | 80.0238 |
| 104.5 | 80.3198 |
| 95 | 80.5325 |
| 85.5 | 80.0967 |
| 76 | 80.8816 |
| 66.5 | 81.4236 |
| 57 | 81.6754 |
| 47.5 | 79.0463 |
| 38 | 77.6362 |
| 28.5 | 73.3266 |
| 19 | 73.0955 |
| 9.5 | 72.2187 |
| 0 | 67.6325 |

***Figure A1. Olivine transects from core to rim (all from tephra samples)***

### Clinopyroxene

Clinopyroxene shows a wide range of compositions and textures. Most are unzoned, with Mg# 65–80 (En40-43Fs12-16Wo42-45); some are slightly normally zoned, from Mg# 80 to Mg#70–75, and a few others are reverse-zoned towards higher Mg# (Mg#45-60 to Mg#70-80). The reverse-zoned group shows significant variations in Mg#, however, and some crystals show oscillatory zoning. We take these reverse-zoned crystals to be xenocrysts, as their cores show significant signs of resorption (rounding), with rims consistent with the erupted magma and the rims of the normally zoned crystals. Figure A2 shows a typical clinopyroxene zoning pattern for the majority of the phenocrysts.

***Figure A2. Typical clinopyroxene zoning pattern. Reverse-zoned xenocryst cores are shown in navy and green.***

The average composition of the unzoned phenocrysts is En42Wo14Fs44. Al_2_O_3_ content is around 5-6wt% in these samples, and TiO_2_ is around 2.3 to 2.5 wt%. The Mg#-poor xenocryst cores, on the other hand, are around 4wt% Al_2_O_3_ and down to 1.0wt% TiO_2_. The full average composition is given in the accompanying spreadsheet.

### Fe-Ti oxides

Whereas both ilmenite and magnetite are present in the samples as phenocrysts, microlites are almost exclusively magnetite in the matrix. Chromite is also present, usually mantled onto olivine (in more primitive compositions) with a magnetite rim. Oxide phenocrysts typically show signs of disequilibrium in the lavas. Ilmenites typically zone to lower TiO_2_ at the rim though some zone to higher TiO_2_ (typically both finally have rims of TiO_2_ ~49wt%). Magnetites show slight zonation in Fe_2_O_3_/FeOt, with a slightly lower ratio nearer the rims (sometimes with a slight oscillation to a higher ratio close to the rim). Examples of magnetite transects are shown in Figure A3. The magnetite phenocryst compositions are very variable, with TiO_2_ ranging from 14-24wt% and the higher values (>20 wt% TiO_2_) are found in the lavas.

| Distance from rim (µm) | Fe2O3/FeO |
| --- | --- |
| 194.79 | 0.786668 |
| 180.92 | 0.769885 |
| 167.05 | 0.782438 |
| 153.18 | 0.784352 |
| 139.31 | 0.779041 |
| 125.44 | 0.768614 |
| 111.57 | 0.777628 |
| 97.7 | 0.774259 |
| 83.83 | 0.780758 |
| 69.96 | 0.769919 |
| 56.09 | 0.76883 |
| 42.22 | 0.772908 |
| 28.35 | 0.758036 |
| 14.48 | 0.752794 |
| 0.61 | 0.717758 |

| Distance from rim (µm) | Fe2O3/FeO |
| --- | --- |
| 450 | 0.757771 |
| 434.5 | 0.752859 |
| 419 | 0.747429 |
| 403.5 | 0.763654 |
| 388 | 0.755163 |
| 372.5 | 0.754284 |
| 357 | 0.75865 |
| 341.5 | 0.756411 |
| 326 | 0.755418 |
| 310.5 | 0.767796 |
| 295 | 0.761242 |
| 279.5 | 0.756089 |
| 264 | 0.760009 |
| 248.5 | 0.761953 |
| 233 | 0.767346 |
| 217.5 | 0.770347 |
| 202 | 0.760297 |
| 186.5 | 0.769182 |
| 171 | 0.764171 |
| 155.5 | 0.767692 |
| 140 | 0.764833 |
| 124.5 | 0.763783 |
| 109 | 0.762306 |
| 93.5 | 0.76013 |
| 78 | 0.753361 |
| 62.5 | 0.735471 |
| 47 | 0.735651 |
| 31.5 | 0.714801 |
| 16 | 0.698666 |
| 0.5 | 0.676028 |

***Figure A3. Magnetite transects from core to rim.***

### Apatite

Fluoro-apatite is present, with two discernable populations. One population contains ~600ppm sulphur, with increasing sulphur and iron content from core to rim. The other population contains no sulphur and has higher SiO_2_. Apatite is found only in batch 2.

### Plagioclase

Plagioclase phenocrysts are mainly normally zoned (Figure A4), with cores An 69-85 and rims of 50-60 (with the exception of some small, normally zoned microphenocrysts that have cores An 35-55 are largely found in lava flows). There are a few reverse-zoned plagioclase phenocrysts with cores from An 35 to An 55 and which are exclusively from batch 2. There are also a number of unzoned phenocrysts, which range in An content from 55 to 83. These are found in all samples (Figure A5).

***Figure A4. Transects across normally zoned plagioclase phenocrysts.***

Plagioclase microlites are abundant and have cores An 33-62, with minor normal zonation to rims An 29-57.

***Figure A5. Feldspar compositions. Note one unstable anorthoclase xenocryst and its rim to the bottom of the plot.***

## Additional H_2_O-CO_2_ models

We first compare our XH_2_O from our SIMS measurements with a thermodynamic model, based on (i) a tephrite composition (Behrens et al, 2009) and (ii) a MORB composition (Dixon et al, 1995; Figure A6).

**Figure A6.** Comparison of XH_2_O (final-fluid) calculated from the SIMS measurements against two thermodynamic models. Green circles show the tephrite (model 1) and yellow triangles show the MORB (model 2) composition.

Figure A6 shows that for lower values of XH_2_O, the thermodynamic models are somewhat less effective, which we interpret is partly due to the production of water through reaction of ferric iron in the starting glass with H_2_ loaded to the vessel such that even very dry charges do end up bearing water. This reaction is enhanced in low XH_2_O conditions due to lower fO_2_. For CO_2_, the data fall between the two models but closer to MORB, consistent with the intermediate composition at Nabro.

We further compared the results of these experiments with two solubility models: that of Papale et al., (2006) and Magmasat (Ghiorso and Gualda, 2015). The result of the comparison is shown in Figure A7. This suggests that for this alkaline composition, Magmasat is a good model for this magma at lower pressures, but underestimates the pressure at higher pressures.

**Figure A7**. Comparison of experimental pressure vs calculated pressures with two models.

The plot below shows a comparison of our data with the model of Iacono-Marziano, 2012.

**Figure A8. Comparison of experimental results with the model of Iacono-Marziano et al., 2012. Line shows 1:1 correlation.**

Figure A8 shows that this model reproduces the experimental results at 100 MPa but not at higher pressures.

## Assessing the temperature model (equation 6)

Figure A9 shows the model derived for our data in equation 6, compared to experimental temperatures.

**Figure A9. Comparison of modelled and predicted temperatures, based on equation 6 in the MS.**
